# Exosome‐transmitted miR‐769‐5p confers cisplatin resistance and progression in gastric cancer by targeting CASP9 and promoting the ubiquitination degradation of p53

**DOI:** 10.1002/ctm2.780

**Published:** 2022-05-06

**Authors:** Xinming Jing, Mengyan Xie, Kun Ding, Tingting Xu, Yuan Fang, Pei Ma, Yongqian Shu

**Affiliations:** ^1^ Department of Oncology The First Affiliated Hospital of Nanjing Medical University Nanjing China; ^2^ Department of Molecular Cell Biology & Toxicology Center for Global Health School of Public Health Nanjing Medical University Nanjing China; ^3^ Jiangsu Key Lab of Cancer Biomarkers Prevention and Treatment Collaborative Innovation Center for Cancer Personalized Medicine Nanjing Medical University Nanjing China

**Keywords:** CASP9, cisplatin resistance, gastric cancer, miR‐769‐5p, p53

## Abstract

**Background:**

Cisplatin resistance is the main cause of poor clinical prognosis in patients with gastric cancer (GC). Yet, the exact mechanism underlying cisplatin resistance remains unclear. Recent studies have suggested that exocrine miRNAs found in the tumor microenvironment participate in tumor metastasis and drug resistance.

**Methods:**

Exosomes isolated from BGC823 and BGC823/DDP culture medium were characterized by transmission electron microscopy and differential ultracentrifugation, and miRNA expression profiles of BGC823 and BGC823/DDP cells derived exosomes were analyzed using miRNA microarray. In vivo and in vitro assays were used to identify roles of exosomal miR‐769‐5p and clarify the mechanism of exosomal miR‐769‐5p regulated the crosstalk between sensitive and resistant GC cells.

**Results:**

In this study, we found that cisplatin‐resistant GC cells communicated with the tumor microenvironment by secreting microvesicles. MiR‐769‐5p was upregulated in GC tissues and enriched in the serum exosomes of cisplatin‐resistant patients. The biologically active miR‐769‐5p could be integrated into exosomes and delivered to sensitive cells, spreading cisplatin resistance. Underlying cellular and molecular mechanism was miR‐769‐5p targeting CASP9, thus inhibiting the downstream caspase pathway and promoting the degradation of the apoptosis‐related protein p53 through the ubiquitin‐proteasome pathway. Targeting miR‐769‐5p with its antagonist to treat cisplatin‐resistant GC cells can restore the cisplatin response, confirming that exosomal miR‐769‐5p can act as a key regulator of cisplatin resistance in GC.

**Conclusions:**

These findings indicate that exosome‐transmitted miR‐769‐5p confers cisplatin resistance and progression in gastric cancer by targeting CASP9 and promoting the ubiquitination degradation of p53. These findings reveal exosomal miR‐769‐5p derived from drug‐resistant cells can be used as a potential therapeutic predictor of anti‐tumor chemotherapy to enhance the effect of anti‐cancer chemotherapy, which provides a new treatment option for GC.

## Graphical abstract

- MiR‐769‐5p was up‐regulated in GC tissues and enriched in the serum exosomes of cisplatin‐resistant patients.
- The biologically active miR‐769‐5p could be integrated into exosomes and delivered to sensitive cells, spreading cisplatin resistance.
- Exosomal miR‐769‐5p could target CASP9, thus inhibiting the downstream caspase pathway.
- Exosomal miR‐769‐5p could promote the degradation of the apoptosis‐related protein p53 through the ubiquitin‐proteasome pathway.

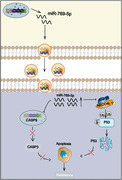

## 1 INTRODUCTION

Gastric cancer (GC) is one the most common malignancies in cancer‐related death worldwide.^1^ Cisplatin has been widely applied to treat patients with advanced metastatic GC not eligible for surgery.^2^ However, as not all patients respond to cisplatin, this may lead to a poor prognosis.^3^ Tumour resistance reveals a complex dynamic process of mutual influence between individuals and tumours. Several factors, such as reduction in drug influx, improvement in drug efflux or metabolism, increase in DNA repair, activation of pro‐survival signaling and inhibition of pro‐apoptotic pathways could determine the development of drug resistance. At the micro‐level, it results from the mutual adaptation of the tumour microenvironment and tumour cells after chemotherapy.^4^ The adaptive changes of tumour cells occur orderly under the control of intricate signal networks and key molecules, where the interaction of heredity, epigenetics and post‐translational protein modification has an important role.

Exosomes are membranous vesicles containing DNA, RNA, protein and other information molecules that are released into the extracellular matrix after the fusion of multi‐vesicles in the cytoplasm and the cell membrane.^5^, ^6^ Exosomes can be used as potential natural carriers to deliver functional proteins, glycans, lipids, metabolites, RNA and DNA to tumour cells to play a biological role.^7^ For example, tumours can release signal over long distances to sites of future metastases to promote formation of a hospitable, pre‐metastatic niche (PMN) to foster growth of disseminated tumour cells upon their arrival.^8^ Exosomes are currently of immense interest for their ability to regulate metastasis by transporting and transferring bioactive molecules between cellular communications because exosomes can dock and fuse to the membrane of target cells and deliver exosomal surface proteins and cytoplasm without eliciting adverse immune responses and possessing low risk for tumour formation. Now, there is a tremendous interest in utilising exosomes as in vivo delivery vehicles for microRNAs (miRNAs). Growing evidence indicates that exosomes released by cancer cells are enriched in miRNAs.^9^, ^10^ Exosomal miRNAs can mediate phenotypical changes in the tumour microenvironment (TME) to promote tumour growth and therapy resistance.^11^ MiRNA is a non‐coding RNA with a length of 18–22 nt, which regulates protein expression levels by blocking mRNA translation or inducing mRNA degradation.^12^ It can modify the expression of target genes and regulate signal transduction and biological processes.^13^ Changes in the expression of certain miRNAs in most tumours have been associated with tumour cell proliferation, angiogenesis and drug resistance.^14^, ^15^ Still, the roles of exosomal miRNAs communicating between cells to confer cisplatin resistance are poorly understood. In this study, we investigated the contributions of miRNAs in cisplatin resistance, mainly focused on miR‐769‐5p, and explored the therapeutic implications for cisplatin‐resistant GC patients. The difference analysis of miR‐769‐5p was conducted through the TCGA database. Literatures reported that miR‐769‐5p has a role in the tumour as an oncogenic miRNA in hepatocellular carcinoma, osteosarcoma, glioma and so on.^16^, ^17^, ^18^, ^19^ However, the role of miR‐769‐5p in cisplatin resistance in GC has not been reported. Therefore, the purpose of this study was to explore the relationship and mechanism between miR‐769‐5p and cisplatin resistance in GC.

The apoptotic signaling molecule CASP9 is one of the caspases, a family of proteins that regulates cell death.^20^, ^21^ Anti‐apoptosis is an important feature of malignant cells, which has been clearly related to tumour development and cancer resistance.^22^ Targeting anti‐apoptosis is considered to be a valuable strategy to improve susceptibility to apoptosis and the response to chemotherapy.^23^, ^24^, ^25^ Another well‐known molecule involved in apoptosis is p53, which can prevent abnormal cell proliferation, canceration and therapy resistance.^26^, ^27^ Ubiquitin–proteasome system (UPS) is a specialised proteolytic system that controls protein degradation and has an important role in cellular protein homeostasis. Evidence supports that up to 80% of cellular proteins are degraded by the UPS, including p53.^28^, ^29^, ^30^, ^31^, ^32^ Based on the above, we wanted to investigate whether miR‐769‐5p is involved in the apoptosis pathway and how to regulate apoptosis‐related proteins in GC cisplatin resistance. We clarified the phenomena via up‐regulation or down‐regulation of miR‐769‐5p directly in GC. Therefore, our results supported the hypothesis that the level of miR‐769‐5p in exosomes could be used as a potential biomarker for evaluating cisplatin resistance in GC.

## 2 MATERIALS AND METHODS

All the materials and methods and abbreviations are included in Supplementary Materials and Methods.

## 3 RESULTS

### 3.1 miR‐769‐5p is enriched in BGC823/DDP cell‐derived exosomes

**FIGURE 1:**
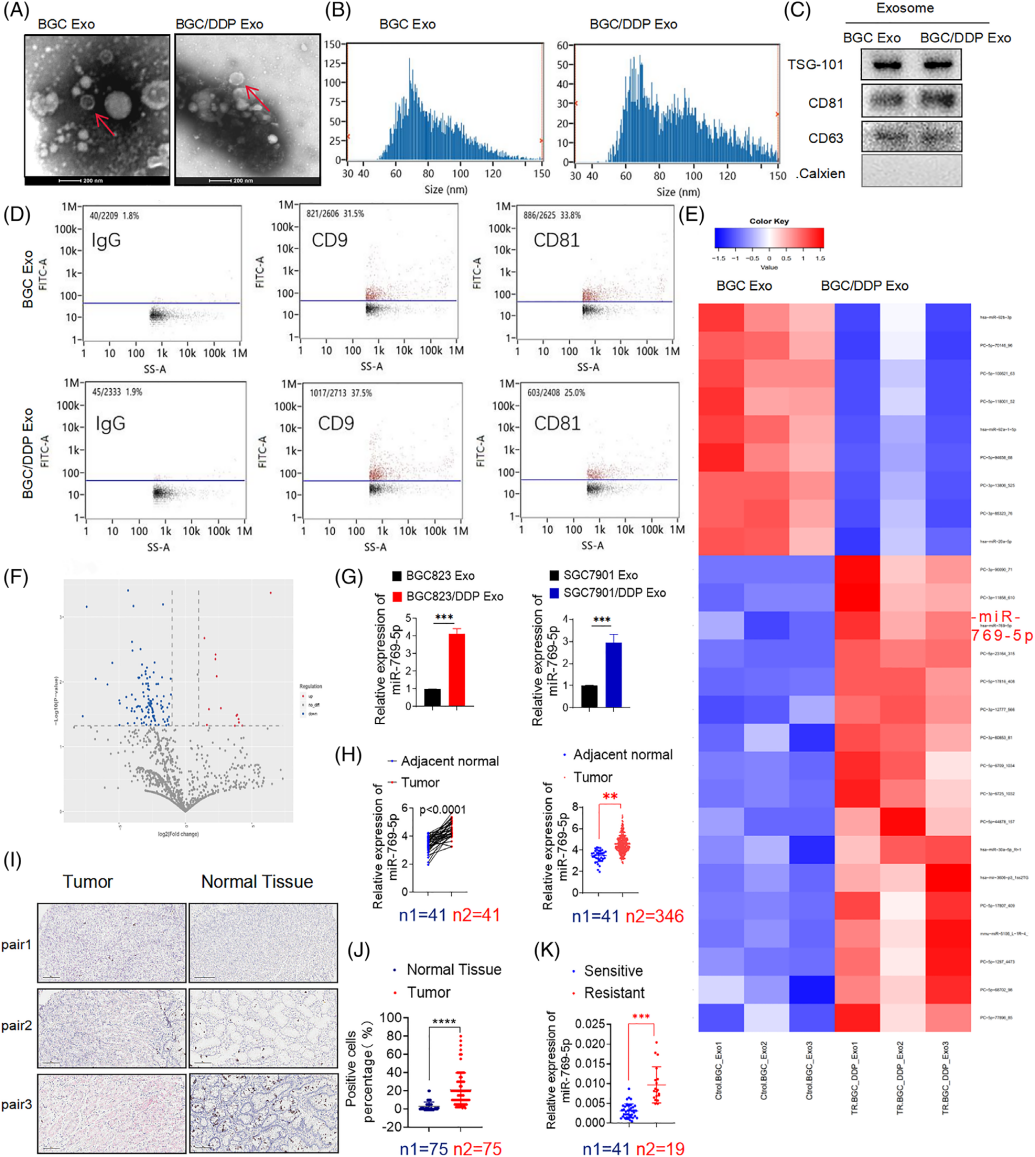
miR‐769‐5p is enriched in BGC823/DDP cell‐derived exosomes. (A) Double‐membrane exosomes purified from the supernatants of BGC823 and BGC8231/DDP cells were observed by transmission electron microscopy (TEM). (B) NanoSight particle tracking analysis (NTA) of the diameter and concentration of vesicles(particles/ml). (C and D) Exosomal markers TSG101, CD9, CD81 and CD63 were detected by Western blot and FCM to prove that the extract in exosomal protein purified from cell supernatants has the typical characteristics of exosomes. (E and F) Cluster heat map and Volcano plot of differential miRNAs in exosomes purified from the supernatants of BGC823 and BGC823/DDP cells. (G) qRT‐PCR verified the relative expression of miR‐769‐5p in exosomes purified from the supernatants of BGC823, BGC823/DDP, SGC7901 and SGC7901/DDP cells. (H) Different expression of miR‐769‐5p between 41 pairs of tumour and adjacent tumour, 41 tumours and 346 adjacent tumours according to TCGA database. (I and J) The positive rate (referring to the percentage of positive cells with red staining) of miR‐769‐5p in 75 pairs of gastric cancer tissues and adjacent tissues by RNA in situ hybridisation (ISH). (K) qRT‐PCR detected the relative expression of miR‐769‐5p in serum exosomes of 60 cases (including 41 cisplatin‐sensitive cases and 19 cisplatin‐resistant cases) of GC patients. The level of serum miR‐769‐5p was significantly increased in non‐response patients (*n*1 = 19) compared with response patients (*n*2 = 41). Quantitative data from three independent experiments are shown as the mean ± SD (error bars). *p < .05, **p < .01, ***p < .001 (Student's *t*‐test)

To isolate exosomes from BGC823 and BGC823/DDP cells, we purified the conditioned medium by using differential centrifugations. Under the transmission electron microscope, nanovesicles were seen as a round shape with bilayered membranes, and the diameter distribution of these nanovesicles ranged from 40 to 150 nm for cryopreserved spheres (Figure 1(A)). NanoSight particle tracking analysis of the size distributions and a number of exosomes revealed that the size of main vesicles secreted from BGC823 and BGC823/DDP cells was 82 and 89 nm, respectively (Figure 1(B)). By immunoblotting of lysates from purified nanovesicles and flow cytometry (FCM), the known exosomal markers TSG101, CD9, CD81and CD63 were detected (Figures 1(C) and 1(D)). These results demonstrated that these nanovesicles isolated from BGC823 and BGC823/DDP presented typical characteristics of exosomes.

Next, we compared the differences in miRNAs expressed in two cell‐derived exosome populations by RNA‐seq (Figures 1(E) and 1(F)). Then, we further analysed the expression of top 2 up‐regulated and 3 down‐regulated miRNAs (Figure S1(A)) in TCGA database (Figure S1(C)). Through RNA‐seq, the level of miR‐769‐5p expressed in BGC823/DDP secreted exosomes (BD Exo) was 4.77 times that in BGC secreted exosomes (BC Exo) (Figure S1(A)). By qRT‐PCR, we found that miR‐769‐5p was the most differentially expressed miRNA. The expression of miR‐769‐5p in BD Exo was 8.778 ± 0.6923‐fold greater than in BC Exo (Figure 1(G)). Moreover, according the TCGA database, we found that miR‐769‐5p may be related to cancer promotion in GC (Figure 1(H)). To find out whether miR‐769‐5p associated with tumorigenesis or progression in GC, miR‐769‐5p expression levels were analysed by combining TCGA database, including 346 GC patients. Compared with the matched normal tissues, the expression of miR‐769‐5p was higher in GC tissues on average than in normal tissues (*p* < .0001).

To detect the miR‐769‐5p expression levels in 75 pairs of clinical samples, we used the technique of RNA in situ hybridisation. Our results revealed that miR‐769‐5p had markedly higher expression in tumour tissues compared with paracancerous tissues (Figures 1(I) and 1(J)). The results indicated that the abundance of miR‐769‐5p in GC tissues was much higher than that in matched normal tissues, and the expression of miR‐769‐5p was correlated with advanced TNM stage and poor prognosis (Table 1). Additionally, we investigated the expression level of miR‐769‐5p in human GC serum samples. The miR‐769‐5p expression level was significantly increased in serum exosomes of cisplatin‐resistant patients (*n* = 19, as compared with serum exosomes of cisplatin‐sensitive patients (*n* = 41, including 19 parental serum exosomes and 22 non‐parental serum exosomes) (Figure 1(K)). These findings suggested that miR‐769‐5p may be involved in cisplatin sensitivity.

**TABLE 1:** Correlation of relative miR‐769‐5p expression with the clinicopathological characteristics of 75 patients with gastric cancer

Relationship between miR‐769‐5p expression and clinicopathologic factors of patients with gastric cancer				
Parameter	Number of patients	miR‐769‐5p (low)	miR‐769‐5p (high)	*p* Value (*p < .05)
Sex				
Male	51	28	23	.285
Female	24	10	14	
Age (year)				
<60	60	33	27	.133
≥60	15	5	10	
Tumour size (cm)				
<5	23	16	7	.029
≥5	52	22	30	
Differentiation grade				
Well‐moderate	43	26	17	.049
Poor‐undifferentiation	32	12	20	
T stage				
T1–T2	7	6	1	.051
T3–T4	68	32	36	
Lymph node status				
Negative	23	17	6	.007
Positive	52	21	31	
Distant metastasis				
M0	75	38	37	
M1	0	0	0	
TNM stage				
I–II	31	21	10	.013
III–IV	44	17	27	

### 3.2 miR‐769‐5p is required for GC cisplatin resistance

**FIGURE 2:**
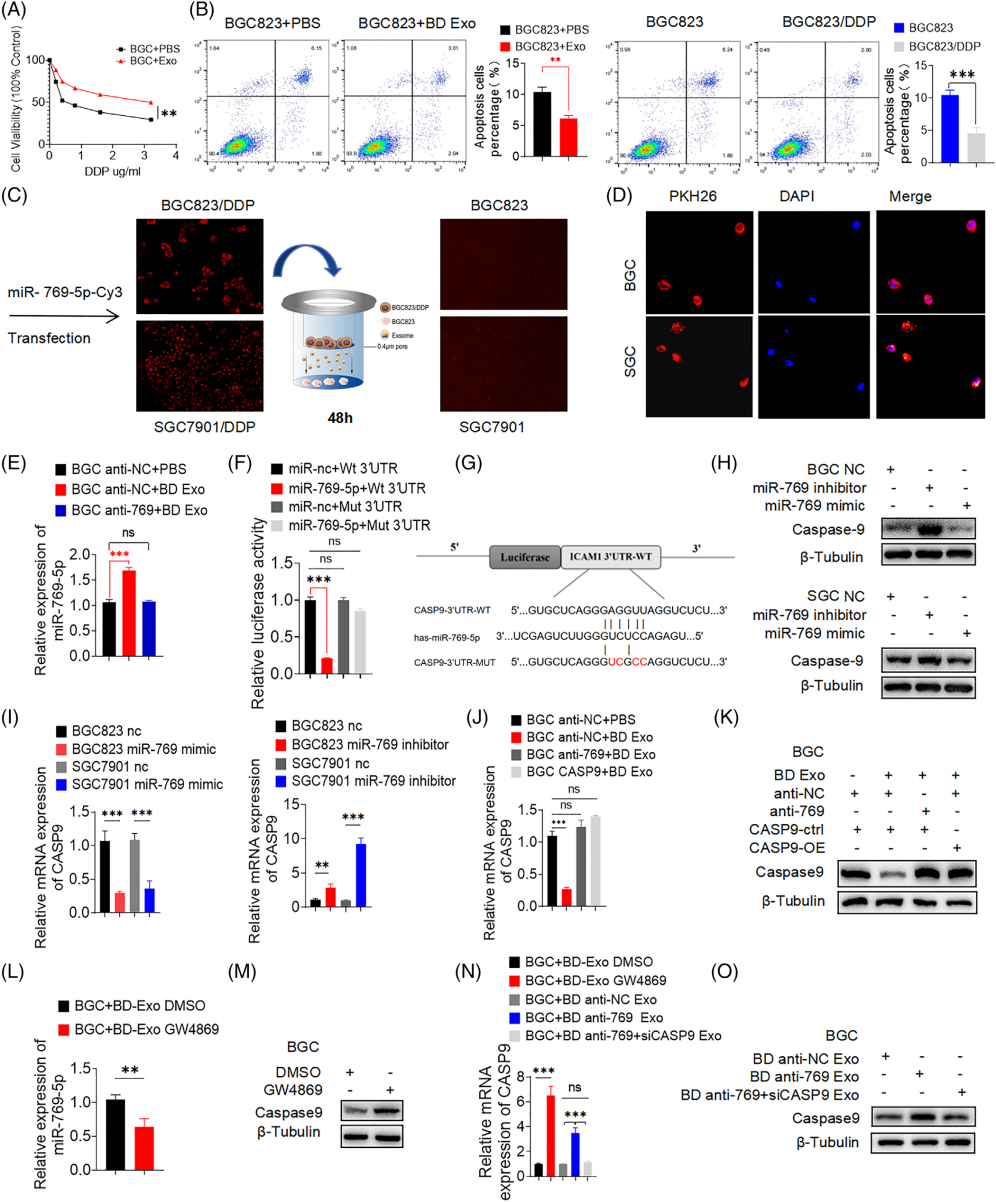
Exosome‐mediated transfer of miR‐769‐5p is required for GC cisplatin‐resistance and targets CASP9 directly. (A) The survival of BGC823 or SGC7901 cells co‐cultured with BD Exo or SD Exo (200 μg/ml) for 24 h and treated with cisplatin for 24 h was detected by CCK‐8. (B) The rates of BGC823 cells’ apoptosis were reduced after being co‐cultured with BD Exo (200ug/ml) for 24 h and treated with cisplatin (0.4 μg/ml) for 24 h detected by FCM. (C) Red fluorescence was observed in the BGC823 or SGC7901 cells after co‐cultured with BGC823/DDP or SGC7901/DDP cells which were transfected with the Cy3‐miR‐769‐5p mimic (red fluorescence) for 48 h. (D) Confocal microscopy showed internalisation of exosomes in BGC823 or SGC7901 recipient cells after co‐cultured with PKH26‐labeled (red fluorescence) BD Exo or SD Exo for 24 h. DAPI was used to stain the nuclei of BGC823 or SGC7901 recipient cells with blue fluorescence. (E) qRT‐PCR showed the expression of miR‐769‐5p in in BGC anti‐NC + PBS, BGC anti‐NC + BD Exo and BGC anti‐769 + BD Exo. (F) Luciferase reporter was carried out in HEK293T co‐transducted with miR‐769‐5p‐mimics or miRNA control with firefly luciferase reporter plasmid containing either wild‐type (WT) or mutant (MUT) CASP9 3′UTR (pGL3‐CASP9‐WT or pGL3‐CASP9‐MUT). (G) Predicted binding sites of the CASP9 3′UTR by miR‐769‐5p. (H and I) PCR and Western blot confirmed that miR‐769‐5p negatively regulated the expression of CASP9. (J and K) The mRNA and protein levels of CASP9 in BGC anti‐NC + PBS, BGC anti‐NC + BD Exo and BGC anti‐769 + BD Exo. (L) qRT‐PCR showed the expression of miR‐769‐5p in BGC + BD Exo DMSO and BGC + BD Exo GW4869. (M and N) qRT‐PCR and Western blot showed the expression of CASP9 in BGC + BD Exo DMSO and BGC + BD Exo GW4869. (N and O) The up‐regulation of CASP9 mRNA and protein was detected by qRT‐PCR and Western blot in BGC + BD anti‐769 Exo. Quantitative data from three independent experiments are shown as the mean ± SD (error bars). *p < .05, **p < .01, ***p < .001 (Student's *t*‐test)

In this study, we hypothesised that miR‐769‐5p from BD Exo might participate in intercellular communication. To verify our hypothesis, we evaluated the effect of cisplatin on BGC823 cells in the presence of BD Exo and found that BD Exo significantly decreased the sensitivity of BGC823 cells to cisplatin by CCK8 (Figure 2(A) and Figure S1(D)). At a cisplatin concentration of 0.8 μg/ml, the survival of BGC823 cells increased after adding BD Exo compared with control. The half‐maximal inhibitory concentration (IC50) of cisplatin was also increased. Additionally, the apoptosis rates of BGC823 cells were reduced after being co‐cultured with BD Exo for 24 h (Figure 2(B)). These data suggested that exosomes secreted from resistant cells could increase IC50 and reduce apoptosis following cisplatin treatment.

A Transwell assay was used to examine whether the delivery of miR‐769‐5p occurs via exosomes. Briefly, we plated BGC823/DDP cells transfected with the Cy3‐miR‐769‐5p mimics in the upper chamber and BGC823 cells in the lower chamber. The co‐culture system was separated by 0.4 μm pores, just allowing the transmission of microparticles, such as exosomes, but inhibiting direct contact between cells. After 48 h, we found strong red fluorescence in BGC823 cells (Figure 2(C)). This phenomenon proved that miR‐769‐5p might be directly transferred from donor cells to recipient cells through exosomes. Furthermore, to visualise exosome transfer, we first incubated BGC823 cells and BD Exo in the presence of PKH26‐labeled for 24 h and evaluated the BD Exo uptake levels by measuring the red PKH26 signal in the BGC823 cell line. The confocal immunofluorescence microscopy detected a robust exosome signal in the cytoplasm of BGC823 cells after incubation of labelled BD Exo (Figure 2(D)), thus suggesting that BD Exo was successfully taken up by BGC823 cells. Figure 2(E) (Figure S1(E)) shows that the co‐incubation with BD Exo increased the expression of miR‐769‐5p.

### 3.3 Exosome‐mediated transfer of miR‐769‐5p targets CASP9 directly

To further explore the mechanism through which BD Exo and miR‐769‐5p induced cisplatin resistance, we investigated the target gene involved in mediating the effect of miR‐769‐5p on modulating apoptosis by TargetScan, MiRWalk and miRTarBase. We found that CASP9 was a target of miR‐769‐5p in 3′‐UTR area. Luciferase reporter assay further showed a significant reduction in luciferase activity when miR‐769‐5p was expressed in HEK293T cells as it did not affect the luciferase activity when the binding site was mutated (Figures 2(F) and 2(G)). Furthermore, qRT‐PCR and Western blot showed that overexpression of miR‐769‐5p inhibited the expression of CASP9 in BGC823 cells, whereas inhibition of miR‐769‐5p reversed this process (Figures 2(H) and 2(I)), thus suggesting that miR‐769‐5p can negatively regulate CASP9 at both the transcript and protein levels.

Next, we infected BGC823 cells with lentiviral vectors to construct cell lines stably expressing miR‐769‐5p inhibitor (BGC anti‐769), negative control miRNA inhibitor (BGC anti‐NC) or CASP9 overexpression (BGC CASP9). Then, we directly cocultured these cells with BD Exo (BGC anti‐769 + BD Exo, BGC anti‐NC + BD Exo and BGC CASP9 + BD Exo). BGC anti‐NC that were incubated with the same amount of PBS (BGC anti‐NC+ PBS) was used as a negative control. Figure 2(E) (Figure S1(E)) shows that the co‐incubation with BD Exo increased the expression of miR‐769‐5p in BGC anti‐NC + BD Exo but had no effect on the BGC anti‐NC + PBS and BGC anti‐769 + BD Exo cells. Compared with the control group BGC anti‐NC + PBS, the expression of CASP9 in BGC anti‐NC + BD Exo was reduced in mRNA and protein level. Nevertheless, when miR‐769‐5p was inhibited in BGC823, the impact above of reduction in CASP9 induced by BD Exo was offset (Figures 2(J), 2(K), S1(F) and S1(G)). These results suggested that BD Exo can induce the up‐regulation of miR‐769‐5p and down‐regulation of CASP9 in recipient cells.

Transwell assay was used to further explore whether the delivery of miR‐769‐5p to recipient cells is dependent on exosomes. We plated BGC823/DDP cells with GW4869 in the upper chamber to prevent exocytosis, whereas BGC823 cells were seeded in the lower chamber. After 24 h, we collected BGC823 cells and found that the expression of miR‐769‐5p in the cells (BGC + BD Exo GW4869) was significantly reduced compared with the control group cells treated with DMSO (BGC + BD Exo DMSO) (Figures 2(L) and S1(H)). The CASP9 mRNA and protein expression were significantly increased (Figures 2(M), 2(N), S1(I) and S1(J)). These results indicated that the delivery of miR‐769‐5p was dependent on exosomes.

In another experiment, we plated BGC823/DDP cells transfected with miR‐769‐5p inhibitor (BD 769 inhibitor) in the upper chamber and BGC823 cells in the lower chamber. We found that the co‐cultured recipient cells CASP9 mRNA (Figures 2(N) and S1(J)) and protein (Figures 2(O) and S1(K)) levels were higher compared with the negative control. In addition, when BD cells in the upper chamber were co‐transfected with anti‐miR‐769‐5p and CASP9‐siRNA (BD anti‐769+siCASP9), exosomes released from BD cells had no statistically significant effect on the mRNA and protein levels of CASP9 in the recipient cells. These results further confirmed that miR‐769‐5p was present in exosomes and that CASP9 was down‐regulated by miR‐769‐5p.

### 3.4 Exosome‐mediated transfer of miR‐769‐5p confers cisplatin resistance through down‐regulating CASP9 and subsequent evasion of apoptosis

**FIGURE 3:**
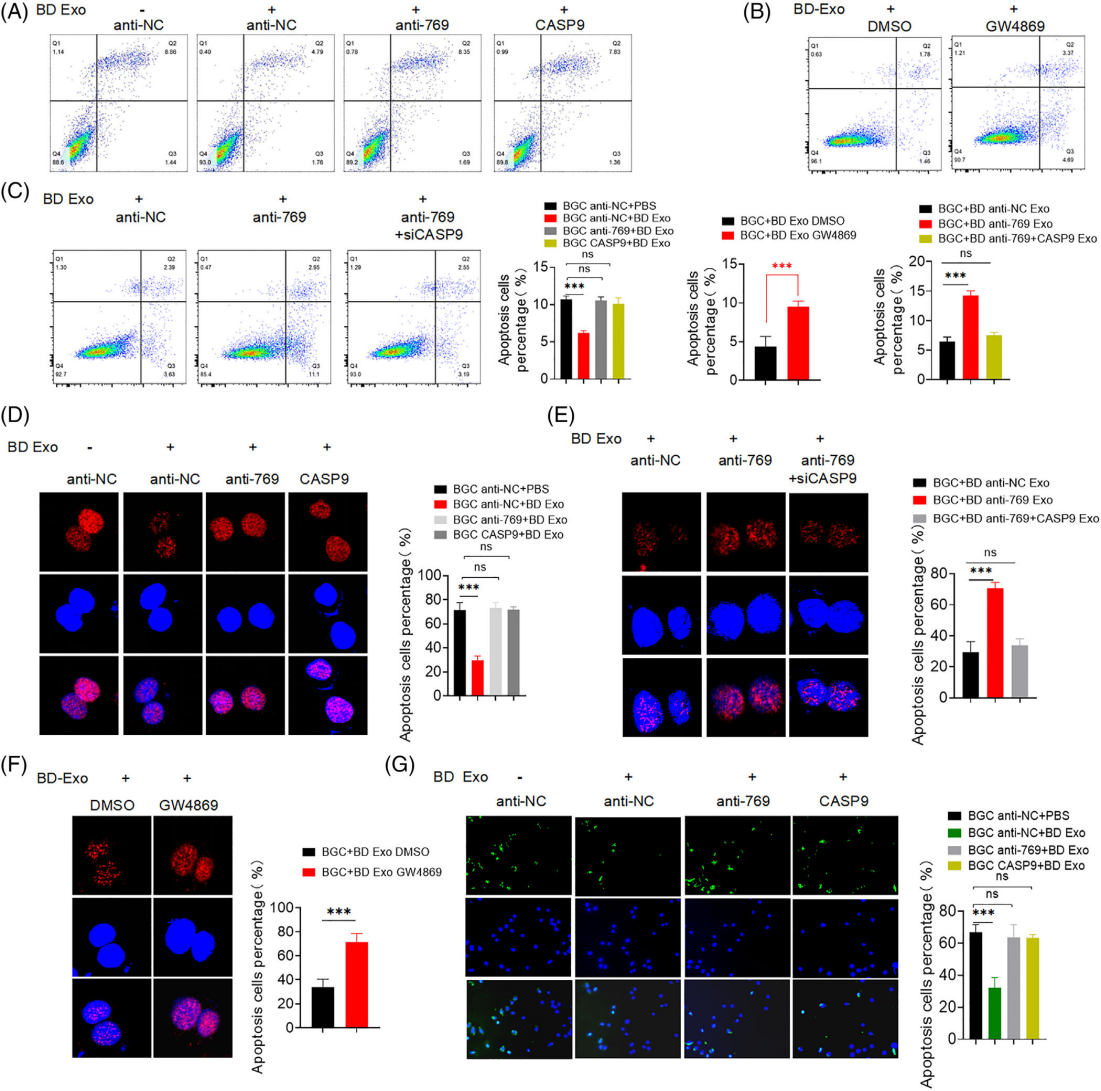
Exosome‐mediated transfer of miR‐769‐5p confers cisplatin resistance through down‐regulating CASP9. (A) FCM assay detected cell apoptosis rate of BGC anti‐NC + PBS, BGC anti‐NC + BD Exo, BGC anti‐769 + BD Exo and BGC CASP9 + BD Exo. (B) FCM assay detected cell apoptosis rate of BGC + BD Exo DMSO and BGC + BD Exo GW4869. (C) FCM assay detected cell apoptosis rate of BGC + BD anti‐NC Exo, BGC + BD anti‐769 Exo and BGC + BD anti‐769+siCASP9 Exo. (D) The level of γ‐H2AX nuclear foci in BGC anti‐NC + PBS, BGC anti‐NC + BD Exo, BGC anti‐769 + BD Exo and BGC CASP9 + BD Exo. (E) The level of γ‐H2AX nuclear foci in BGC + BD anti‐NC Exo, BGC + BD anti‐769 Exo and BGC + BD anti‐769+siCASP9 Exo. (F) The level of γ‐H2AX nuclear foci in BGC + BD Exo DMSO and BGC + BD Exo GW4869. (G) TUNEL analysis detected cell apoptosis rate of BGC anti‐NC + PBS, BGC anti‐NC + BD Exo, BGC anti‐769 + BD Exo and BGC CASP9 + BD Exo. Quantitative data from three independent experiments are shown as the mean ± SD (error bars). *p < .05, **p < .01, ***p < .001 (Student's *t*‐test)

Next, we determined whether exosomal miR‐769‐5p confers cisplatin resistance in BGC823 cells by targeting CASP9. As shown in Figure 3(A) (Figure S2(A)), BD Exo significantly down‐regulated the apoptosis of BC anti‐NC cells induced by cisplatin, whereas no statistically significant difference was observed in BGC823 cells with miR‐769‐5p knockdown or CASP9 overexpression. Therefore, miR‐769‐5p knockdown or CASP9 overexpression in BGC823 could reverse the effect of BD Exo on the cisplatin resistance of BGC823 cells. Compared with BGC823/DDP cells treated with DMSO, after co‐cultivation with BGC823/DDP cells treated with GW4869 (an inhibitor that inhibits exosomes release), the level of apoptosis of BGC823 cells induced by cisplatin was increased (Figures 3(B) and S2(B)). In addition, when they were co‐cultured with miR‐769‐5p knockdown BGC823/DDP cells, the cisplatin resistance of BGC823 cells was decreased (Figures 3(C) and S2(C)).

γ‐H2AX is a sign of DNA double‐strand breaks. Twenty‐four hours after cisplatin treatment, the level of γ‐H2AX nuclear foci in the control group remained high, but the nuclear foci in the BD Exo co‐culture group significantly decreased (Figures 3(D) and S3(A)). However, there was no statistically significant difference observed in BGC823 cells with miR‐769‐5p knockdown or CASP9 overexpression. γ‐H2AX expression levels in nuclear foci indicated that cisplatin induces more resistant cell lines after co‐culturing with BD Exo. Similarly, after co‐culturing with BGC823/DDP cells treated with GW4869, the level of γ‐H2AX expression in nuclear foci of BGC823 cells induced by cisplatin was increased compared with BGC823/DDP cells treated with DMSO (Figures 3(F) and S3(B)). When we co‐cultured BGC823/DDP cells transfected with miR‐769‐5p inhibitor (BD anti‐769) with BGC823 cells seeded in the lower chamber, we found that γ‐H2AX expression levels in nuclear foci of co‐cultured recipient cells were higher than the negative control. Co‐incubation of BGC823/DDP cells co‐transfected with miR‐769‐5p inhibitor and CASP9‐siRNA had no profound synergistic effect on γ‐H2AX expression in BGC823 cells (Figures 3(E) and S3(C)).

**FIGURE 4:**
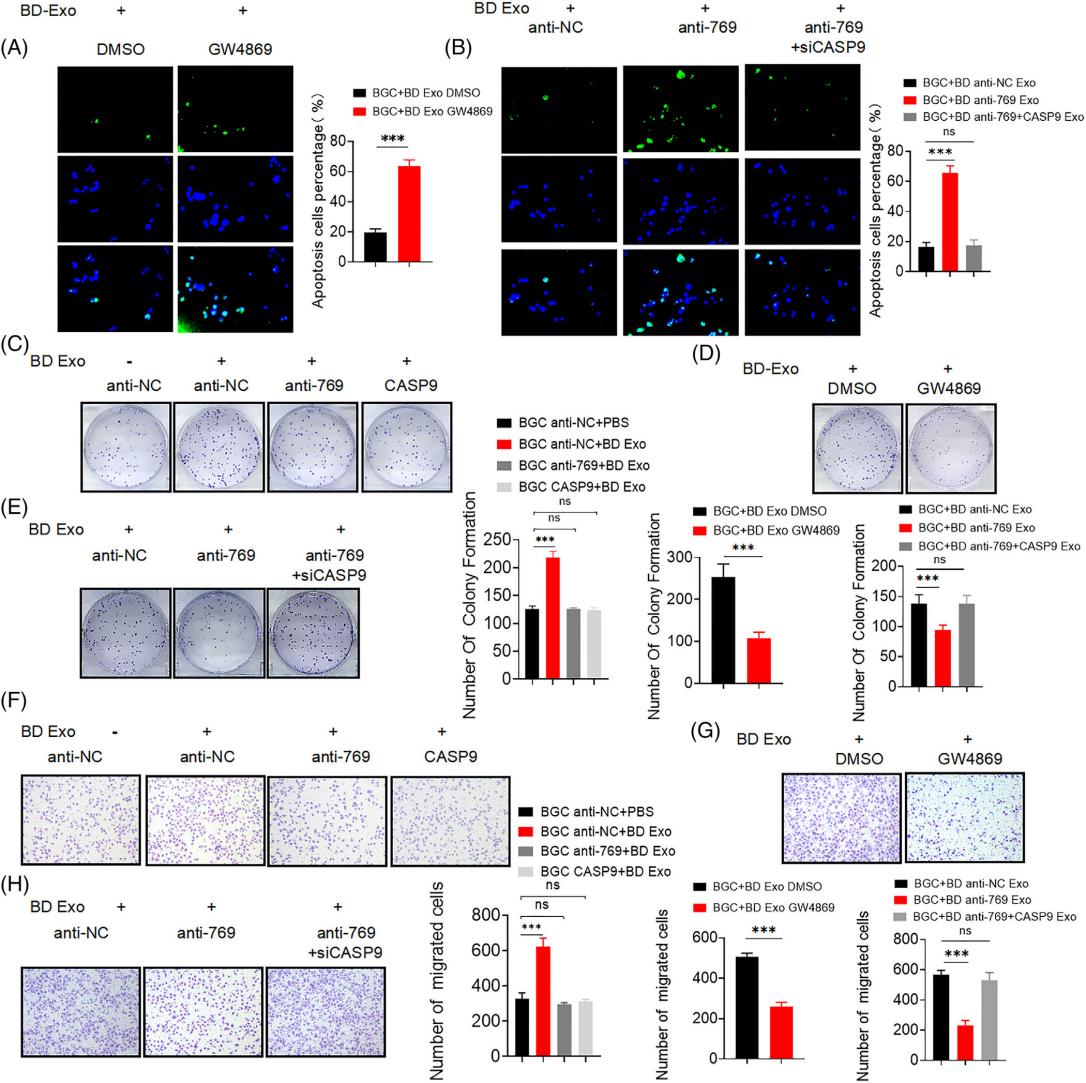
Exosomal miR‐769‐5p promotes recipient cells proliferation and migration by down‐regulating CASP9. (A) TUNEL analysis detected cell apoptosis rate of BGC + BD Exo DMSO and BGC + BD Exo GW4869. (B) TUNEL analysis detected cell apoptosis rate of BGC + BD anti‐NC Exo, BGC + BD anti‐769 Exo and BGC + BD anti‐769+siCASP9 Exo. (C) The average colony numbers of three independent experiments were calculated in BGC anti‐NC + PBS, BGC anti‐NC + BD Exo, BGC anti‐769 + BD Exo and BGC CASP9 + BD Exo. (D) The average colony numbers of three independent experiments were calculated in BGC + BD Exo DMSO and BGC + BD Exo GW4869. (E) The average colony numbers of three independent experiments were calculated in BGC + BD anti‐NC Exo, BGC + BD anti‐769 Exo and BGC + BD anti‐769 + siCASP9 Exo. (F) Migration ability of BGC anti‐NC + PBS, BGC anti‐NC + BD Exo, BGC anti‐769 + BD Exo and BGC CASP9 + BD Exo were assessed by Transwell assay. (G) Migration ability of BGC + BD Exo DMSO and BGC + BD Exo GW4869 were assessed by Transwell assay. (H) Migration ability of BGC + BD anti‐NC Exo, BGC + BD anti‐769 Exo and BGC + BD anti‐769 + siCASP9 Exo were assessed by Transwell assay. Quantitative data from three independent experiments are shown as the mean ± SD (error bars). *p < .05, **p < .01, ***p < .001 (Student's *t*‐test)

**FIGURE 5:**
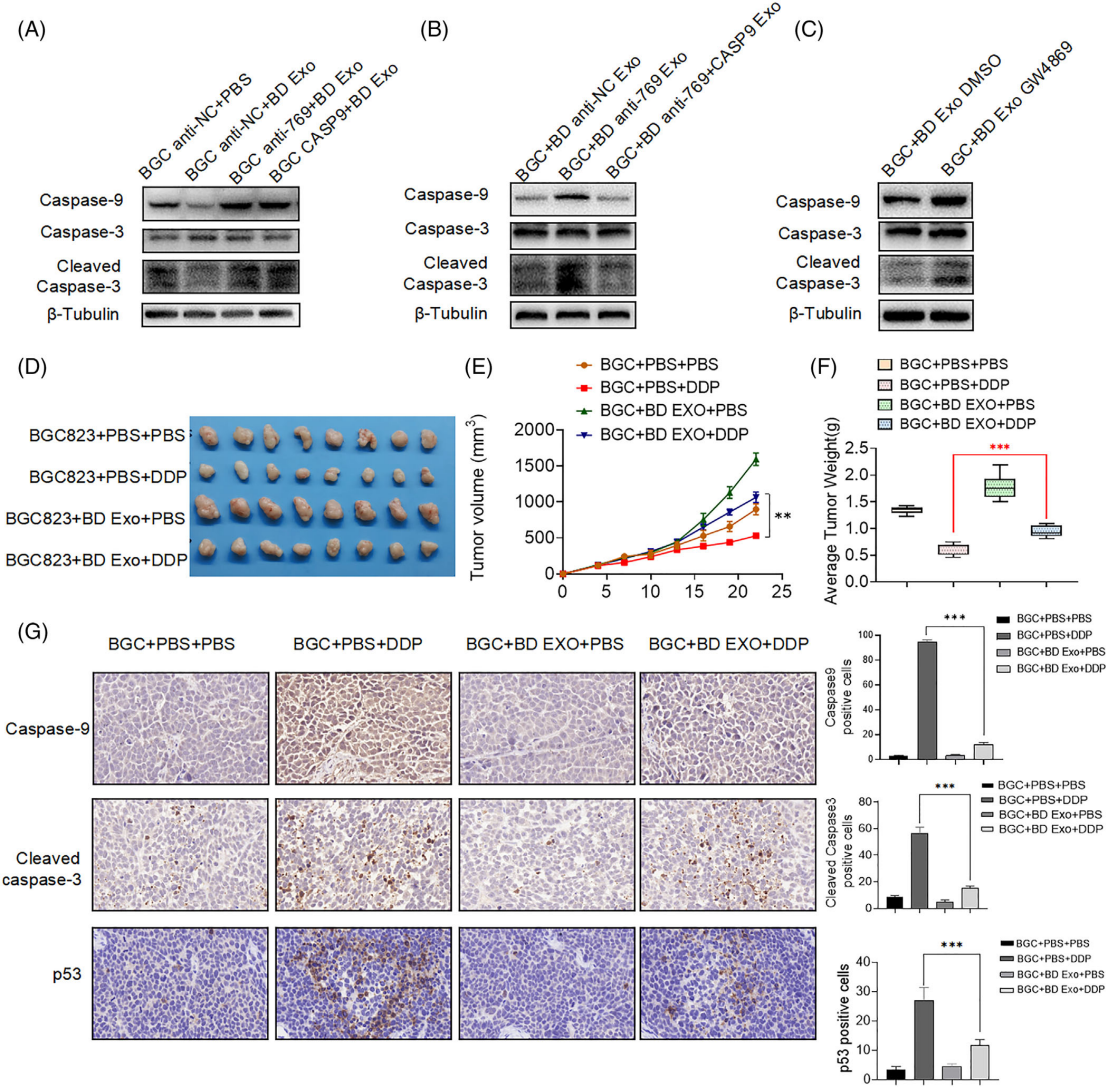
Exosomal miR‐769‐5p confers cisplatin resistance through down‐regulating CASP9 along with subsequent evasion of apoptosis and confirmed in vivo. (A) Western blot analysis of caspase9, caspase3 and cleaved caspase3 in BGC anti‐NC + PBS, BGC anti‐NC + BD Exo, BGC anti‐769 + BD Exo and BGC CASP9 + BD Exo. (B) Western blot analysis of caspase9, caspase3 and cleaved caspase3 in BGC + BD Exo DMSO and BGC + BD Exo GW4869. (C) Western blot analysis of caspase9, caspase3 and cleaved caspase3 in BGC + BD anti‐NC Exo, BGC + BD anti‐769 Exo and BGC + BD anti‐769 + siCASP9 Exo. (D) Subcutaneous xenograft assay of BGC823 cells with or without BD Exo (200 μg/100 μl cells per mouse) once every 2 days in nude mice with PBS or cisplatin (DDP, 4 mg/kg) treatment. (E) Tumour volume of xenograft models were measured every 3 days and shown. Tumour volume (mm^3^) = 0.5 × width^2^ × length. (F) Tumour weight of xenograft models were measured every 3 days and shown. (G) CASP9, cleaved caspase3 and p53 expression levels were shown in representative xenograft tumours by Immunohistochemistry (IHC) (400× magnification, scale bars = 50 μm). Results are presented as mean ± SD. *p < .05, **p < .01, ***p < .001

To further investigate the role of exosomal miR‐769‐5p cisplatin‐induced apoptosis, we performed TUNEL analysis and found that it was consistent with the verification of FCM assays (Figures 3(G)–4(B) and S3(D)–S3(F)). The results showed that the exosomal miR‐769‐5p from cisplatin‐resistant cells could moderate cell apoptosis of cisplatin‐sensitive cells. Western blot demonstrated that the protein levels of caspase‐9 and cleaved caspase‐3 in BGC anti‐NC + BD Exo cells were reduced; yet, there were no obvious differences in the BGC anti‐769 + BD Exo and BGC CASP9 + BD Exo cells (Figures 5(A) and S4(A)). Compared with BGC + BD Exo DMSO or BGC + BD anti‐NC Exo cells, the caspase‐9 and cleaved caspase‐3 protein levels were increased in BGC823 cells co‐cultured with BGC823/DDP cells treated with GW4869 or transfected with miR‐769‐5p inhibitor (Figures 5(B), 5(C), S4(B) and S4(C)). Thus, these data suggested that the knockdown miR‐769‐5p could reverse the chemoresistance of GC cells to cisplatin.

Importantly, in vivo study intra‐tumour injection of BD Exo promoted the growth and induced the cisplatin resistance of GC cells compared with the same group injected with PBS (Figures 5(D)–5(G)). Four‐week‐old BALB/c nude mice were obtained from Model Animal Research Center of Nanjing University, China. All animal studies (including the mice euthanasia procedure) were done in compliance with the regulations and guidelines of Nanjing Medical University institutional animal care and conducted according to the AAALAC and the IACUC guidelines (IACUC‐1902006). As shown in the Figures 5(D) and 5(E), after the equal cisplatin treatment, the tumour volume of BGC + BD Exo + DDP group was larger than that of BGC + PBS + DDP group, indicating that exosomes of drug‐resistant cells can promote tumour growth and reduce the sensitivity to cisplatin. Taken together, we have reasons to believe that miR‐769‐5p might be transferred via exosomes from resistant GC cells to the neighbouring sensitive GC cells, thereby spreading cisplatin resistance.

### 3.5 Exosomal miR‐769‐5p promotes recipient cells proliferation and migration by down‐regulating CASP9

Next, we investigated whether exosomal miR‐769‐5p affects the biological processes of GC cells. BGC anti‐NC cells treated with BD EXO showed increased colony formation, migration capacity compared with BGC anti‐NC cells treated with PBS (Figures 4(C), 4(F), S2(D) and S2(G)). Nevertheless, this alteration was reversed when BGC anti‐769 or BGC CASP9 cells were co‐cultured with BD Exo. In contrast, when BGC823 cells were co‐cultured with BGC823/DDP treated with GW4869 or miR‐769‐5p knockdown, the colony formation, migration capacity of BGC823 cells decreased compared with those of the corresponding negative controls (Figures 4(D)–4(H) and S2(E)–S2(I)). Our findings suggested that exosomal miR‐769‐5p enhanced GC cell proliferation and migration by down‐regulating CASP9.

To sum up, the miR‐769‐5p was markedly up‐regulated in BGC823 cells treated with BD Exo, which suggested its potential role in cisplatin resistance and indicated the possibility of achieving the cisplatin resistance through the exosomal transfer of miR‐769‐5p by targeting CASP9 in GC cells.

### 3.6 miR‐769‐5p promotes ubiquitin‐mediated p53 protein degradation in GC cells

**FIGURE 6:**
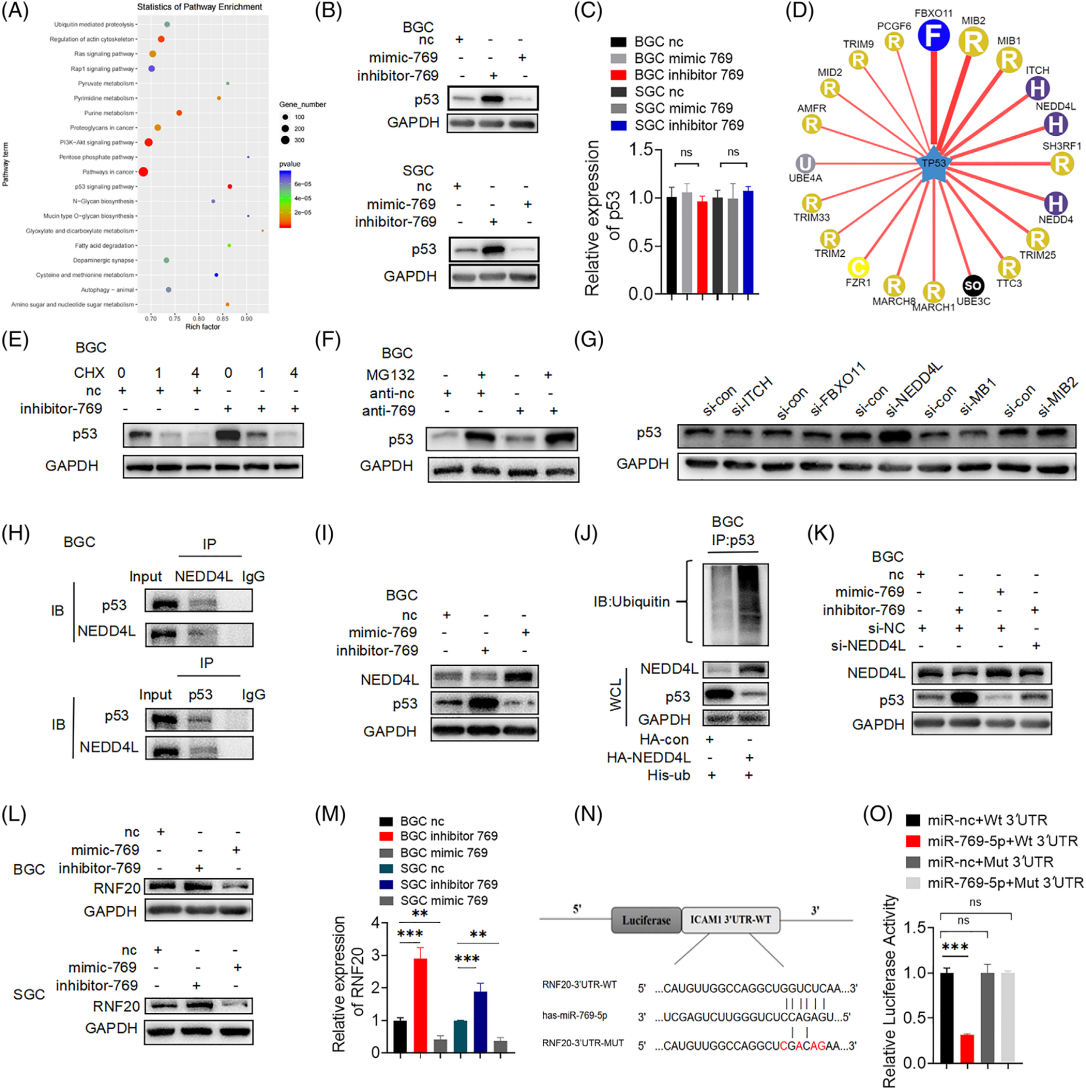
miR‐769‐5p promotes ubiquitin‐mediated p53 protein degradation in GC cells. (A) KEGG enrichment analysis showed that the target genes of differentially expressed miRNAs are enriched in the p53 pathway. (B) Western blot analysis of expression level of p53 protein in in BGC NC, BGC mimic‐769 and BGC inhibitor‐769. (C) qRT‐PCR detected the expression level of p53 mRNA in BGC NC, BGC mimic‐769 and BGC inhibitor‐769. (D) UbiBrowser website predicted E3 ubiquitination ligase with p53 as a substrate. (E) Western blot analysis of p53 protein level of 100 μg/ml treated with cycloheximide (CHX) changes with treatment time (0, 1, 4 h). (F) Analysis of p53 protein level by Western blot in BGC nc and BGC inhibitor‐769 after treatment of MG‐132 (10 μm) for 6 h. (G) Western blot analysis of p53 protein expression level after transfection of E3 ubiquitinated ligase specific small interfering RNA (siRNA): siITCH, siFBXO11, siNEDD4L, siMIB1 and siMIB2. (H) Co‐IP detected the interaction between NEDD4L and p53 in GC cells. (I and K) The expression of NEDD4L and p53 protein levels when miR‐769‐5p is knocked down or overexpressed. (J) Co‐IP and Western blot detected p53 ubiquitination modification mediated by NEDD4L. (L and M) Western blot and qRT‐PCR verified the negatively regulatory effects of miR‐769‐5p on RNF20. (N) Predicted binding sites of the RNF20 3′UTR by miR‐769‐5p. (O) Luciferase reporter was carried out in HEK293T co‐transducted with miR‐769‐5p‐mimics or miRNA control with pGL3‐RNF20‐WT or pGL3‐RNF20‐MUT. Quantitative data from three independent experiments are shown as the mean ± SD (error bars). *p < .05, **p < .01, ***p < .001 (Student's *t*‐test)

As is well known, the transcription factor p53 is essential in the complex molecular network regulating apoptosis, cell proliferation and carcinogenesis.^33^, ^34^, ^35^ To further determine whether miR‐769‐5p is involved in GC cisplatin resistance and its molecular mechanism, we found that the targets of differentially expressed miRNAs were enriched in the p53 pathway based on the KEGG enrichment analysis of differently expressed miRNAs in exosomes (Figure 6(A)). Considering the KEGG analysis of miR‐769‐5p (Table S3), we hypothesised that miR‐769‐5p might affect the p53 pathway. To evaluate whether miR‐769‐5p is involved in p53‐mediated apoptosis of GC cells, miR‐769‐5p expression in BGC823 and SGC7901 cells was overexpressed and knocked down by miR‐769‐5p mimics and inhibitors, respectively, after which the expressions of p53 mRNA and protein were analysed. Western blotting showed that miR‐769‐5p silencing significantly enhanced the expression of p53 in GC cells, whereas overexpression of miR‐769‐5p had the opposite effects (Figure 6(B)). It indicated that miR‐769‐5p negatively regulated p53 protein expression and p53‐mediated apoptosis in GC cells. However, qRT‐PCR showed that the transcription level of p53 was not affected by miR‐769‐5p, indicating that the p53 protein in GC cells may be degraded by ubiquitination (Figure 6(C)). As a result, we transfected miR‐769‐5p inhibitors into GC cells, and 24 h later, the cells were treated with 20 μg/ml cycloheximide (CHX, a protein synthesis inhibitor). The cell lysates were then collected within a specified time period and analysed by Western blot. In Figure 6(E), p53 protein synthesis was inhibited by CHX treatment for 0, 1 and 4 h. Compared with the miR‐769‐5p inhibitor untreated group, the protein level of p53 in the miR‐769‐5p inhibitor‐treated group increased significantly at each time point with CHX treatment. These results indicated that the degradation of p53 was related to proteasome, and miR‐769‐5p knockdown inhibited the degradation process (Figures 6(E) and S4(D)). We also treated the cells with MG‐132 (a specific inhibitor of a ubiquitin‐binding protein) and found that higher expression of p53 protein was detected in the cells treated with MG‐132 (Figures 6(F) and S4(E)). This indicated that p53 protein degradation depends on ubiquitination.

According to UbiBrowser, we characterised the p53‐specific E3 ubiquitin ligases to determine the mechanism of miR‐769‐5p‐mediated p53 ubiquitination in GC cells (Figure 6(D)). We selected the top five  E3 ubiquitin ligases of p53 to be silenced by sequence‐specific small interfering RNA (siRNA) in HEK‐293T. Detection of p53 protein showed that when NEDD4L expression was knocked down by sequence‐specific siRNA, p53 levels increased (Figure 6(G)). NEDD4L is the key E3 ubiquitin ligase for p53 ubiquitination in GC cells.^36^, ^37^, ^38^ However, the negative control of NEDD4L‐siRNA did not affect p53 expression.

Co‐immunoprecipitation (Co‐IP) and Western blot were used to detect the interaction between NEDD4L and p53 in GC cells (Figures 6(H), 6(I), S4(F) and S4(G)). The NEDD4L overexpression plasmid and His‐Ub plasmid were co‐transfected in BGC, and the ubiquitination level of p53 was detected by immunoprecipitation and Western blotting. NEDD4L overexpression promoted the ubiquitination of p53 (Figures 6(J) and S4(H)), which indicated that NEDD4L mediated the ubiquitination modification. In order to further evaluate the effect of miR‐769‐5p on the expression of NEDD4L, we inhibited and overexpressed miR‐769‐5p in GC cell lines to detect the expression of NEDD4L and p53 protein levels (Figures 6(K) and S4(I)). Compared with the negative control group, knockdown of miR‐769‐5p significantly reduced the expression of NEDD4L and increased the expression level of p53, whereas overexpression of miR‐769‐5p showed the opposite result. Western blot also demonstrated that NEDD4L silencing caused p53 protein accumulation in miR‐769‐5p‐silenced cancerous cells. It suggested that the inhibition of miR‐769‐5p could inhibit the expression of E3 ubiquitinated ligase NEDD4L, increasing the level of substrate p53. Therefore, we speculated that miR‐769‐5p could promote the expression of NEDD4L, leading to its participation in the p53 ubiquitination degradation process.

### 3.7 E3 ubiquitination ligase RNF20 participates in miR‐769‐5p‐mediated p53 protein ubiquitination in GC cells

According to the miRNAs target gene prediction, we found that NEDD4L was not the target gene of miR‐769‐5p. So, it was unclear how miR‐769‐5p regulated the expression of NEDD4L. Based on the miRNA target gene prediction website and UbiBrowser website, we found that E3 ubiquitin ligase RNF20 might be the target gene of miR‐769‐5p (Figures 6(L)–6(O)). To characterise the interaction between miR‐769‐5p and RNF20, a dual‐luciferase reporter assay was conducted in HEK293T cells. The results revealed that compared with the controls, miR‐769‐5p significantly decreased the activity of the reporter luciferase that was fused with the wild‐type RNF20 3′‐untranslated region (UTR) (Figure 6(O)). This observation suggested a direct interaction between miR‐769‐5p and RNF20 mRNA. Reports showed that a low RNF20 level was correlated with shortened overall survival and disease‐free survival, indicating poor prognosis in GC patients.^39^, ^40^

**FIGURE 7:**
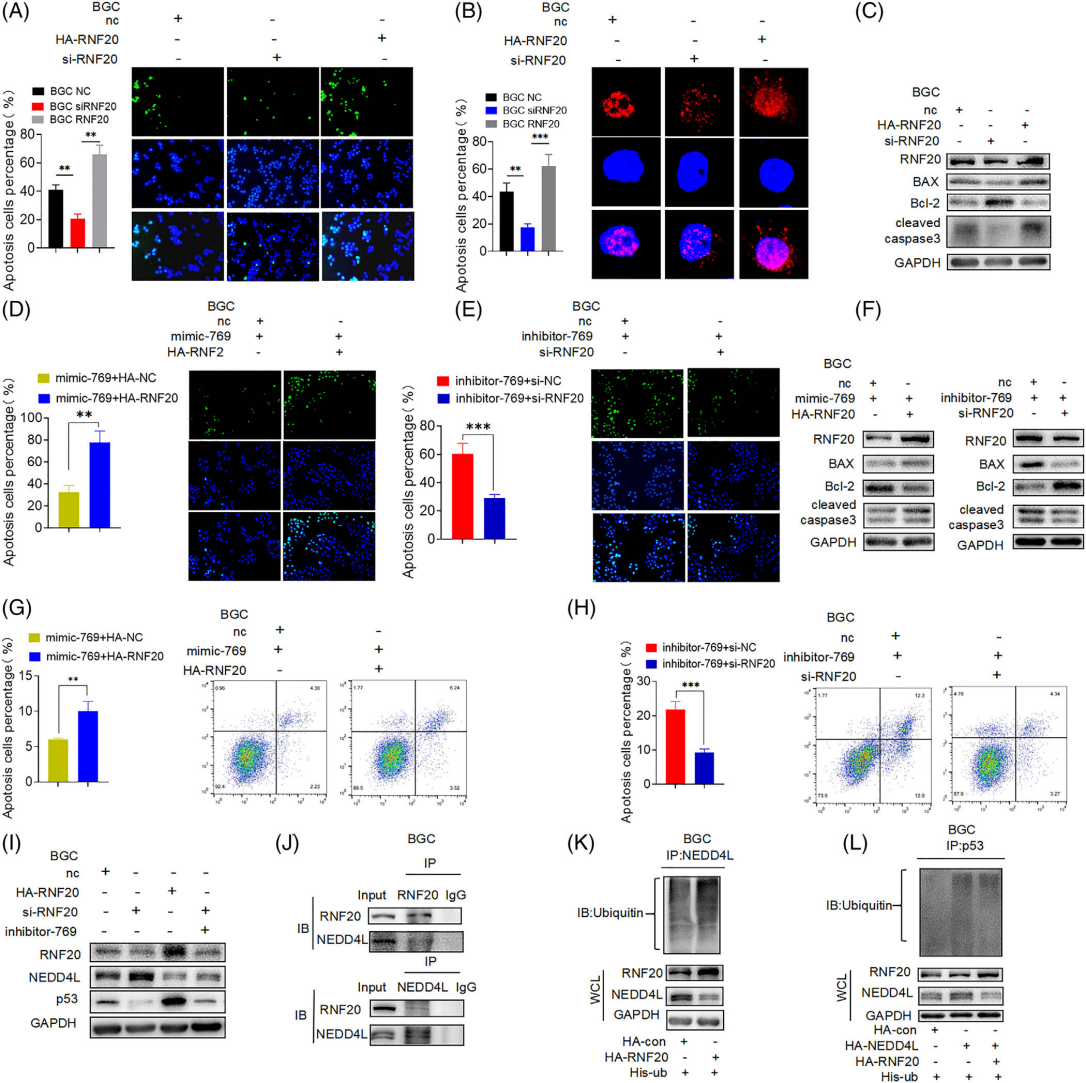
E3 ubiquitination ligase RNF20 participates in miR‐769‐5p‐mediated p53 protein ubiquitination in GC cells. (A) TUNEL analysis detected cell apoptosis rate of BGC NC, BGC si‐RNF20 and BGC HA‐RNF20. (B) The level of γ‐H2AX nuclear foci in BGC NC, BGC si‐RNF20 and BGC HA‐RNF20. (C) The Western blot analysis of Bax, Bcl‐2 and cleaved caspase 3 proved the positive mediation of RNF20 on apoptosis. (D–F) The recovery proved that miR‐769‐5p inhibits the process of apoptosis by down‐regulating RNF20 by analysis of TUNEL and Western blot. (G and H) FCM assay proved that miR‐769‐5p inhibits the process of apoptosis by down‐regulating RNF20. (I) The protein levels of NEDD4L and p53 when RNF20 overexpression and knockdown. (J) Co‐IP proves that NEDD4L interacts with RNF20. (K and L) Co‐IP proves that the ubiquitination modification of NEDD4L is mediated by RNF20. Quantitative data from three independent experiments are shown as the mean ± SD (error bars). *p < .05, **p < .01, ***p < .001 (Student's *t*‐test)

Additionally, we discovered that RNF20 and NEDD4L interacted in GC cells. We transfected silenced or overexpressed RNF20 and negative control plasmids in BGC823, then tested the effect of RNF20 on apoptosis. TUNEL assay showed that compared with the negative control group, overexpression of RNF20 significantly promoted the apoptosis in GC while inhibition of RNF20 conversely (Figures 7(A) and S5(A)). Correspondingly up‐regulated or down‐regulated miR‐769‐5p can reverse this effect (Figures 7(D)–7(F) and S5(C)–S6(B)). The observation in the immunofluorescence of γ‐H2AX were consistent with the TUNEL assay (Figures 7(B) and S5(B)). Moreover, overexpressed RNF20 resulted in up‐regulation of cleaved caspase 3 and activated apoptosis (Figures 7(C) and S6(A)). And the activation of apoptosis influenced by RNF20 was further confirmed by FCM assay (Figures 7(G) and 7(H)). Above results indicate that RNF20, as a target gene of miR‐769‐5p, can participate in cell apoptosis.

As RNF20 has been determined as a target gene of miR‐769‐5p to participate in cell apoptosis, we further figured out how RNF20 conveys apoptotic signals in p53‐mediated cell apoptosis. The gene expression of RNF20 was silenced or overexpressed in GC cells, followed by RNF20 and p53 protein detection. RNF20 overexpression markedly suppressed NEDD4L expression and simultaneously induced p53 expression in GC cells (Figures 7(I) and S6(C)), whereas silenced RNF20 had the opposite effect on NEDD4L and p53 expression in GC. Furthermore, we overexpressed the RNF20 plasmid in GC cells and performed Co‐IP with anti‐RNF20 to identify proteins that interacted with RNF20. Our results indicated that RNF20 was bound to NEDD4L (Figures 7(J) and S6(D)), suggesting RNF20 participated in p53‐mediated GC apoptosis by regulating NEDD4L expression.

To clarify whether NEDD4L could be ubiquitinated by RNF20 (Figures 7(K) and S6(E)), the His‐Ub and RNF20 were co‐expressed in GC cells, and anti‐NEDD4L were used to pull down modified proteins. The presence of polyubiquitinated NEDD4L was observed as a smeared band because of the heterogeneous modification of this protein. At the same time, we stained the polyubiquitinated NEDD4L in the flag‐Ub immunoprecipitants to confirm that the ubiquitination modification of NEDD4L was mediated by RNF20 and found that RNF20 overexpression further enhanced the polyubiquitinated NEDD4L compared with the control (Figures 7(L) and S6(F)). These findings revealed that RNF20 was an E3 ligase for NEDD4L and that RNF20 polyubiquitinated NEDD4L for degradation.

### 3.8 Exosomal miR‐769‐5p induces cisplatin resistance and promotes the tumorigenesis of GC in vivo

**FIGURE 8:**
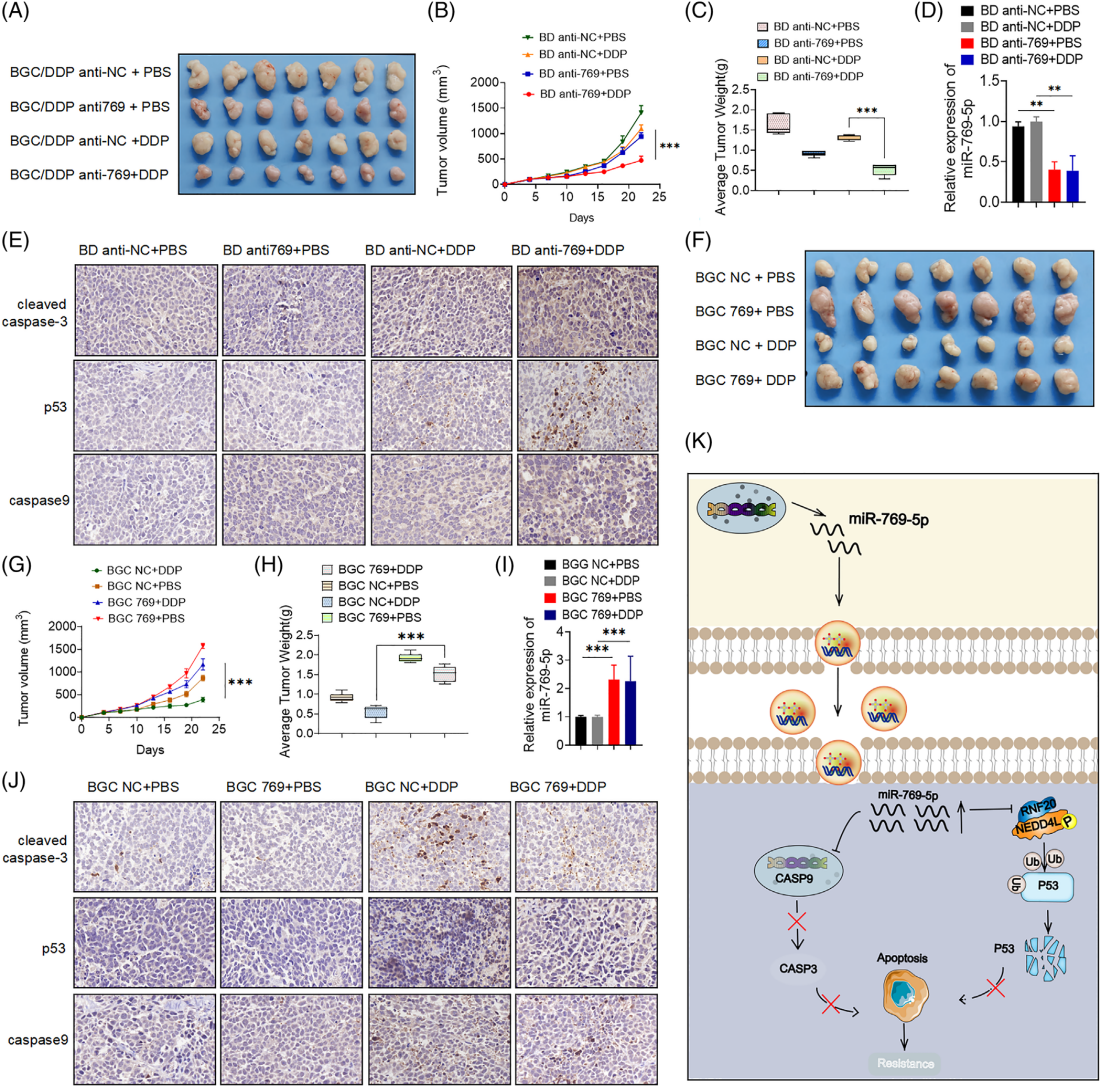
Exosomal miR‐769‐5p induces cisplatin resistance and promotes the tumorigenesis of GC in vivo. (A) Subcutaneous xenograft assay of BGC823/DDP cells (5 × 10^6^ cells/100 μl) with or without miR‐769‐5p knockdwon in nude mice with PBS or cisplatin (DDP, 4 mg/kg) treatment. (B) Tumour volume of xenograft models were measured every 3 days and shown. (C) Tumour weight of xenograft models were measured every 3 days and shown. (D) Levels of exosomal miR‐769‐5p in the serum were detected by qPCR. (E) CASP9, cleaved caspase3 and p53 expression levels were shown in representative xenograft tumours by immunohistochemistry (IHC) (400× magnification, scale bars = 50 μm). (F) Subcutaneous xenograft assay of BGC823 cells (5 × 10^6^ cells/100 μl) with or without miR‐769‐5p overexpressed in nude mice with PBS or cisplatin (DDP, 4 mg/kg) treatment. (G) Tumour volume of xenograft models were measured every 3 days and shown. (H) Tumour weight of xenograft models were measured every 3 days and shown. (I) Levels of exosomal miR‐769‐5p in the serum were detected by qPCR. (J) Caspase9, cleaved caspase3 and p53 expression levels were shown in representative xenograft tumours by Immunohistochemistry (IHC) (400× magnification, scale bars = 50 μm). (K) Summary of the mechanism by which exosomal miR‐769‐5p induces cisplatin resistance. Results are presented as mean SD. *p < .05, **p < .01, ***p < .001

Given the observed effects of exosomal miR‐769‐5p on GC cells in vitro, we subsequently confirmed the aforementioned results in vivo. To determine whether miR‐769‐5p sensitises GC cells to chemotherapeutic agents in vivo, anti‐miR‐769‐5p‐transfected BGC823/DDP cells were subcutaneously implanted into nude mice and then treated with cisplatin. Our data indicated that miR‐769‐5p knockdown significantly decreased cisplatin resistance in BGC823/DDP xenografts (Figures 8(A)–8(C)). Levels of exosomal miR‐769‐5p were approximately two folds higher in the serum compared with the negative control group (Figure 8(D)). The expression levels of CASP9, p53 and cleaved caspase3 were increased when the level of miR‐769‐5p decreased in the subcutaneous tumour tissues of mice (Figure 8(E)). These data provided support for our hypothesis that knockdown miR‐769‐5p ameliorates cisplatin‐resistant GC in vitro and in vivo.

In addition, we subcutaneously injected the stably transfected BGC 823 NC and BGC823 769 cells into nude mice and found that the tumours of BGC823 769 grew faster than those of BGC823 NC. After cisplatin treatment, the tumour volume of the BGC823 769 group was significantly higher compared with BGC 823 NC group (Figures 8(F)–8(J)). These results indicated that miR‐769‐5p could promote growth and induce the cisplatin resistance of BGC823 cells in vivo. Collectively, miR‐769‐5p expression was indispensable for cisplatin resistance in GC cells.

## 4 DISCUSSION

Chemotherapy is the most important treatment for patients who cannot undergo surgery or those with advanced metastatic GC.^41^ Yet, multi‐drug resistance, which has been associated with a poor prognosis, remains a major hurdle to long‐lasting survival of patients with GC.^3^ For example, cisplatin resistance presents a big obstacle in treating patients with advanced GC, which can be partly explained by the highly dynamic spread of cancer cells.

miRNAs can be encapsulated in exosomes to avoid degradation. Exosomal miRNA can be transported to recipient cells and change their phenotype through changes in gene expression.^42^, ^43^, ^44^ For example, drug‐resistant cancer cells may release exosomal miRNAs into the microenvironment, causing the recipient cells to develop drug resistance.^45^, ^46^, ^47^ This ability of exosomes shed from tumour‐resistant cells to transfer drug‐resistant phenotypes to drug‐sensitive cells is considered an important mechanism of drug resistance that is mainly spread through drug efflux pumps and miRNAs’ transfer. Therefore, exosomes might have an important role in invasive tumour progress and chemotherapy resistance.

Our results showed that miR‐769‐5p in exosomes derived from cisplatin‐resistant cells could confer drug‐resistant phenotypes on recipient cells and alter their gene expression and apoptosis. However, transfection of anti‐miR‐769 into BD cells partially blocked the effect of BD Exo on cisplatin. These results indicated that the delivery of miR‐769‐5p was dependent on exosomes. Figure 8(K) summarises the mechanism through which drug‐resistant cells transfer miR‐769‐5p‐loaded exosomes to sensitive cells and modulated cisplatin resistance. Mechanistically, exosomal miR‐769‐5p inhibits cell apoptosis by targeting the downstream caspase pathway of CASP9 inactivation and enhancing the drug resistance of recipient cells to cisplatin (Figure 8(K)).

The activation of the tumour suppressor p53 is essential to prevent abnormal cell proliferation and canceration. Many studies have shown that p53 is involved in the regulation of drug resistance. For example, phosphorylation of p53 serine 15 (Ser15) and serine 20 (Ser20) has been identified as essential in cisplatin resistance.^48^, ^49^ As a key cellular protein regulator, ubiquitination can cause protein degradation. In the process of protein ubiquitination, E3 ubiquitin ligase determines substrate specificity and substrate selection. In addition, the mechanism of ubiquitin‐mediated p53 protein degradation has been extensively studied.^50^, ^51^ For example, mdm2‐dependent p53 polyubiquitination and degradation can regulate cell proliferation, DNA damage‐induced apoptosis and tumorigenesis by inhibiting p53.^52^, ^53^ However, the role of miRNA in the regulation of p53 protein ubiquitination remains unclear.

Looking for the target genes of miR‐769‐5p, we found that miR‐769‐5p promotes the degradation of p53 and inhibits apoptosis through the ubiquitin–proteasome pathway, thus promoting the resistance of GC cells to cisplatin. Our study revealed a new mechanism of p53 protein ubiquitination mediated by miR‐769‐5p in cisplatin resistance. As an important apoptosis‐related protein, miR‐769‐5p participates in the apoptosis of GC cells through the RNF20‐NEDD4L‐p53 pathway in the process of induced apoptosis, and miR‐769‐5p can directly inhibit the expression of RNF20. Previous studies have shown that HBRE1 /RNF20 is the E3 ubiquitin ligase of hiprotein H2B, and the deletion of RNF20 as a tumour suppressor can lead to the overall decrease of H2Bub level.^54^, ^55^ Our results proved that RNF20 had a critical role in p53 protein ubiquitination in GC cells, mediating the direct degradation of p53 protein by E3 ubiquitin ligase NEDD4L, thus revealing a novel miRNA‐mediated p53 protein ubiquitination pathway.

As a complex genetic disease, chemotherapy and radiation therapy of cancer have always been the core treatment options. However, these measures have adverse side effects. Due to malignant tumours being highly heterogeneous in their occurrence and development, regardless of the molecular expression profile or signal network characteristics, tumour cells in different parts of the same tumour may hugely differ. In this context, highly expressed miR‐769‐5p in cisplatin‐resistant cells can be taken up by cisplatin‐sensitive recipient cells through exosomal transfer, thereby decreasing the sensitivity of the recipient cells to cisplatin and providing new ideas for the clinical treatment of cisplatin resistance based on tumour heterogeneity. The pathways for regulating tumour drug resistance are complex, and the crosstalk between many pathways is important for the development of drug resistance. Many previous studies have emphasised that exosomes have a key role in tumour biological characteristics by affecting the tumour microenvironment, which emphasises the importance of considering the cell and tissue environment when studying the key pathways of drug resistance. Herein, we discussed the role miR‐769‐5p has in exosomes as a key determinant of cisplatin resistance in GC. However, further studies are needed to fully understand the biology of exosomes. Current research has highlighted a series of exosome‐dependent pathways, which can be used for the study of targeted therapies for tumour development, metastasis and drug resistance. Many unanswered questions regarding the role of exosomes and their contents in regulating established resistance also remain to be addressed.

## 5 CONCLUSION

Our results proved that miR‐769‐5p, which is highly expressed in drug‐resistant GC cells, can be transferred to recipient cells sensitive to cisplatin via exosomes by targeting the downstream caspase pathway of CASP9 inactivation and mediating the direct degradation of p53 protein by E3 ubiquitin ligase NEDD4L. The specific induction of GC cell apoptosis and cisplatin resistance indicates that inhibiting miR‐769‐5p may represent a potential therapeutic intervention strategy for treating refractory GC.

## CONFLICT OF INTEREST

The authors declare that they have no conflict of interest.

## Supporting information



Figure S1

Figure S2

Figure S3

Figure S4‐S5

Figure S6

Figure S7‐S8

Supporting Information

Supporting Information
