# Supplementary material for: Exosome‐transmitted miR‐769‐5p confers cisplatin resistance and progression in gastric cancer by targeting CASP9 and promoting the ubiquitination degradation of p53
Source: Clin Transl Med. 2022 May 6;12(5):e780. doi: 10.1002/ctm2.780 (PMC9076018; doi:10.1002/ctm2.780)
Supplement: Supplementary file 1 — Figure S1 [file CTM2-12-e780-s001.pdf]

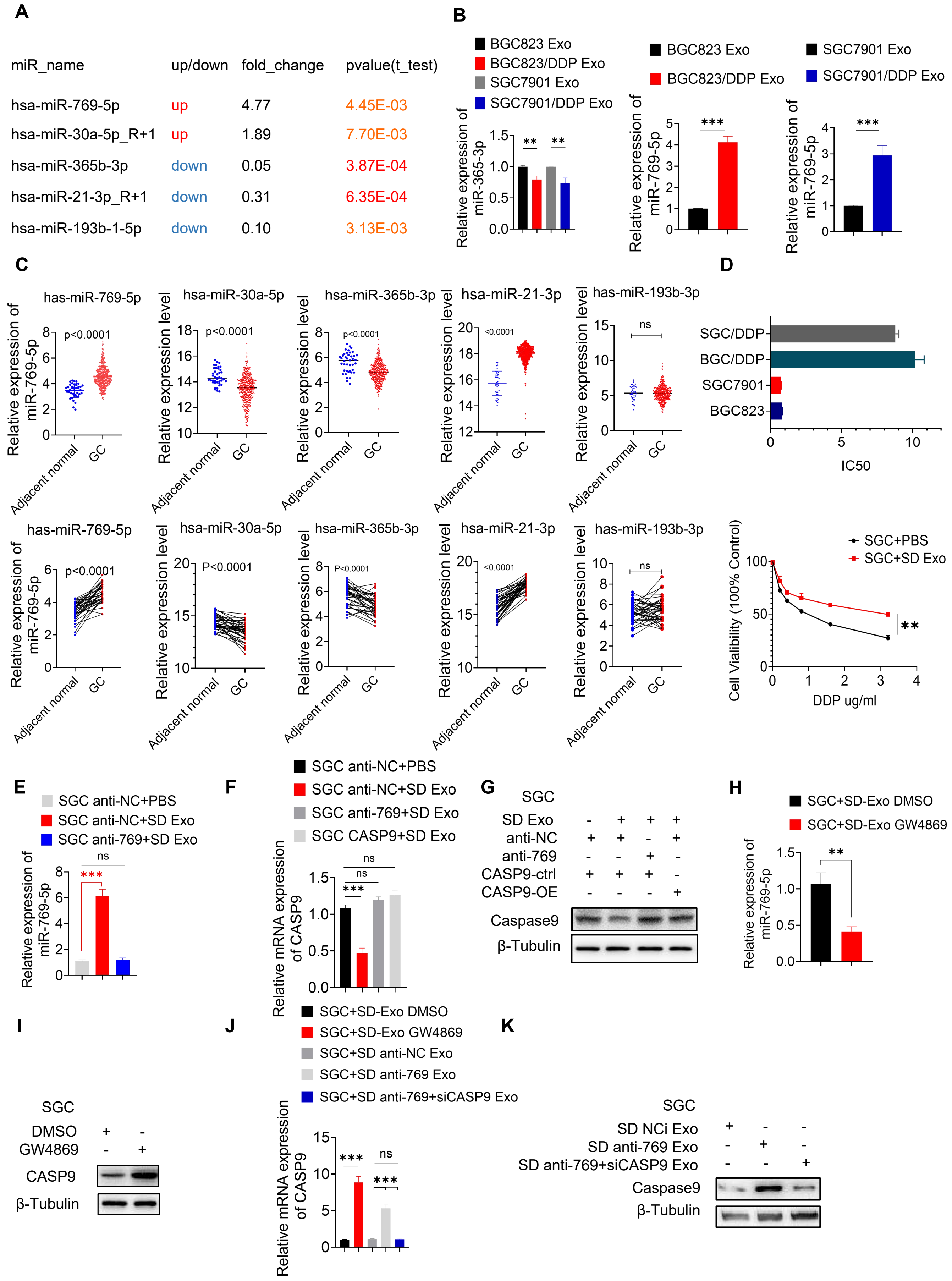
A
miR_name up/down fold_change pvalue(t_test)
hsa-miR-769-5p up 4.77 4.45E-03
hsa-miR-30a-5p_R+1 up 1.89 7.70E-03
hsa-miR-365b-3p down 0.05 3.87E-04
hsa-miR-21-3p_R+1 down 0.31 6.35E-04
hsa-miR-193b-1-5p down 0.10 3.13E-03
B
BGC823 Exo
BGC823/DDP Exo
SGC7901 Exo
SGC7901/DDP Exo
Relative expression of miR-365-3p
Relative expression of miR-769-5p
C
has-miR-769-5p
hsa-miR-30a-5p
hsa-miR-365b-3p
hsa-miR-21-3p
has-miR-193b-3p
p<0.0001
ns
Relative expression level
Adjacent normal
GC
D
SGC/DDP
BGC/DDP
SGC7901
BGC823
IC50
SGC+PBS
SGC+SD Exo
Cell Viability (100% Control)
DDP ug/ml
E
SGC anti-NC+PBS
SGC anti-NC+SD Exo
SGC anti-769+SD Exo
F
SGC anti-NC+PBS
SGC anti-NC+SD Exo
SGC anti-769+SD Exo
SGC CASP9+SD Exo
Relative mRNA expression of CASP9
G
SGC
SD Exo
anti-NC
anti-769
CASP9-ctrl
CASP9-OE
Caspase9
β-Tubulin
H
SGC+SD-Exo DMSO
SGC+SD-Exo GW4869
I
SGC
DMSO
GW4869
CASP9
β-Tubulin
J
SGC+SD-Exo DMSO
SGC+SD-Exo GW4869
SGC+SD anti-NC Exo
SGC+SD anti-769 Exo
SGC+SD anti-769+siCASP9 Exo
K
SGC
SD NCi Exo
SD anti-769 Exo
SD anti-769+siCASP9 Exo
Caspase9
β-Tubulin
