# Supplementary figures and images for: Exosome‐transmitted miR‐769‐5p confers cisplatin resistance and progression in gastric cancer by targeting CASP9 and promoting the ubiquitination degradation of p53
Source: Clin Transl Med. 2022 May 6;12(5):e780. doi: 10.1002/ctm2.780 (PMC9076018; doi:10.1002/ctm2.780)

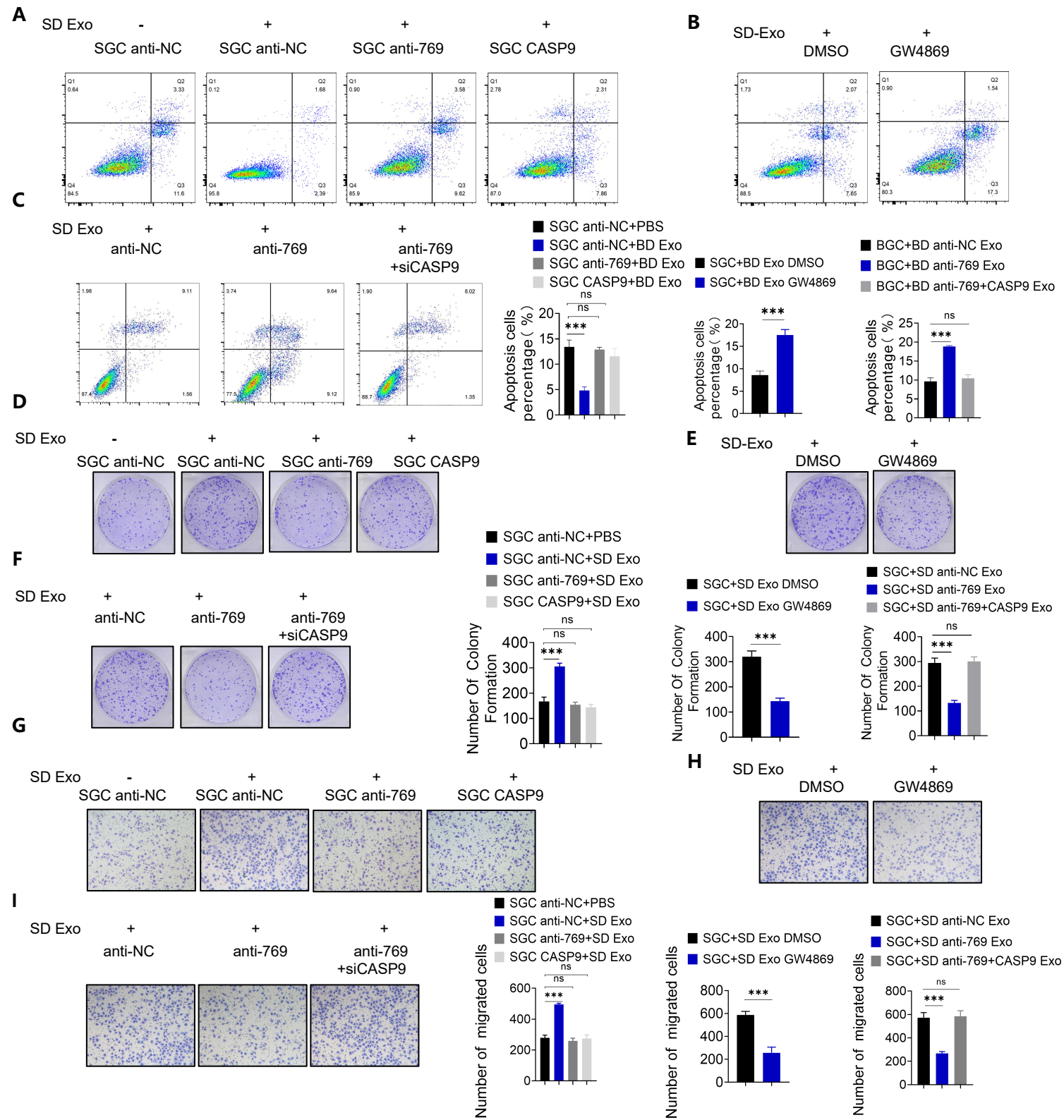

Supplement: Supplementary file 2 — Figure S2 [file CTM2-12-e780-s003.pdf]

**A**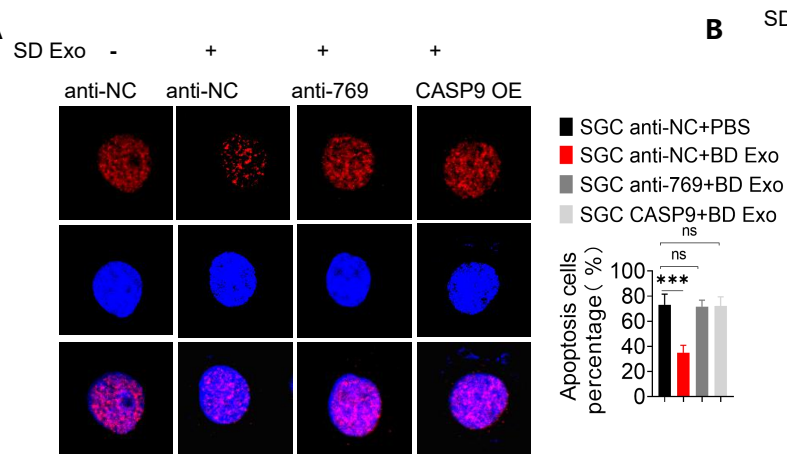**B**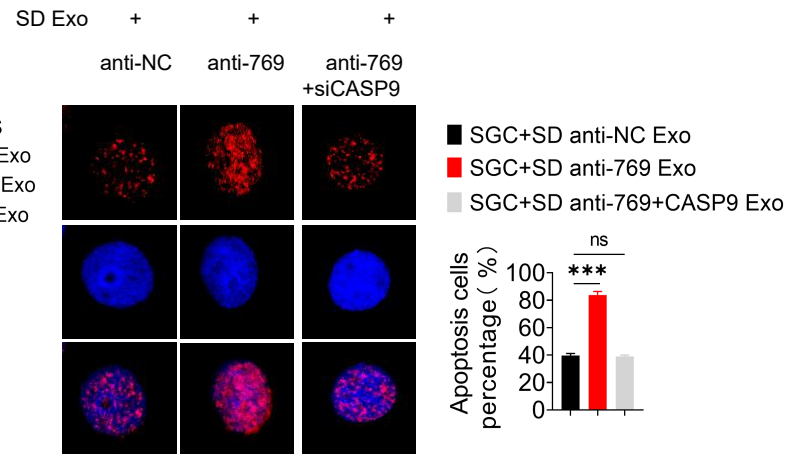**C**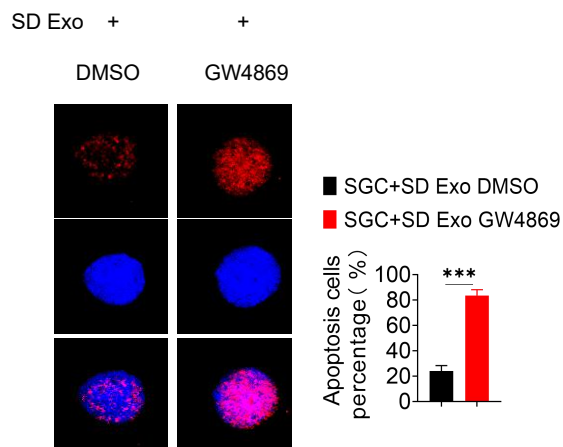**D**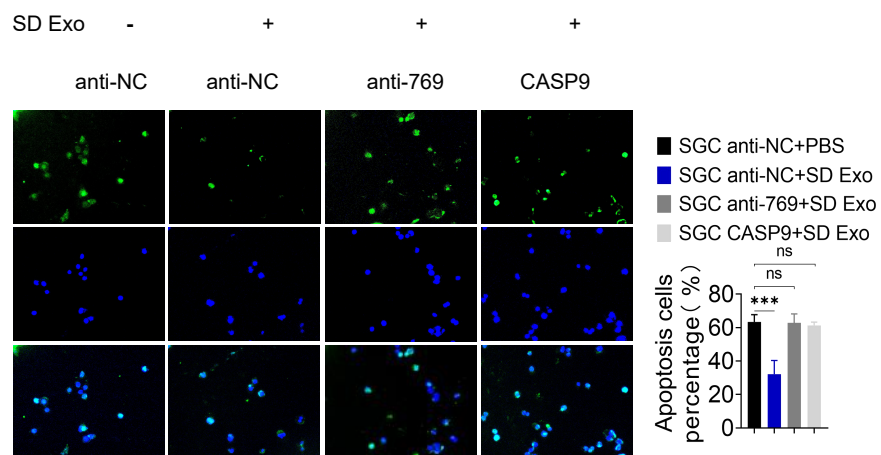**E**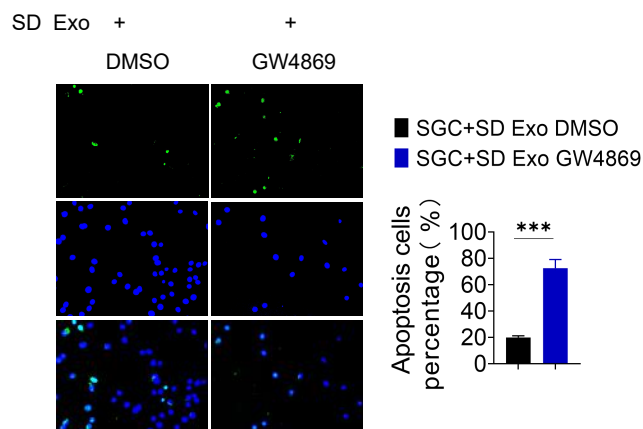**F**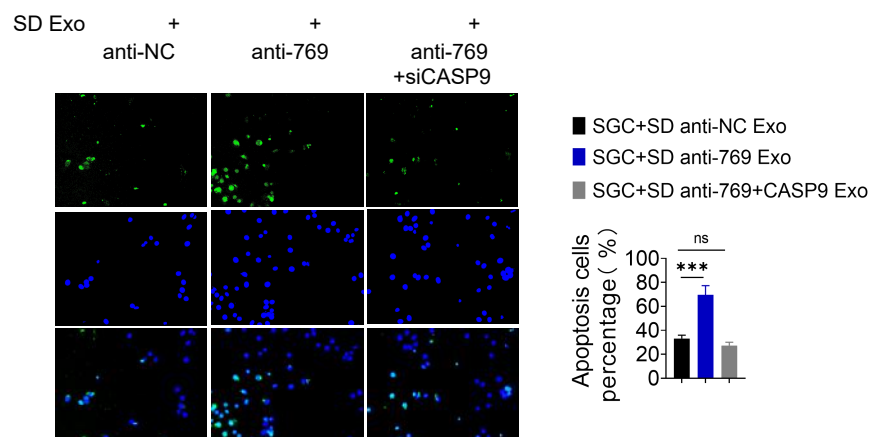

Supplement: Supplementary file 3 — Figure S3 [file CTM2-12-e780-s005.pdf]

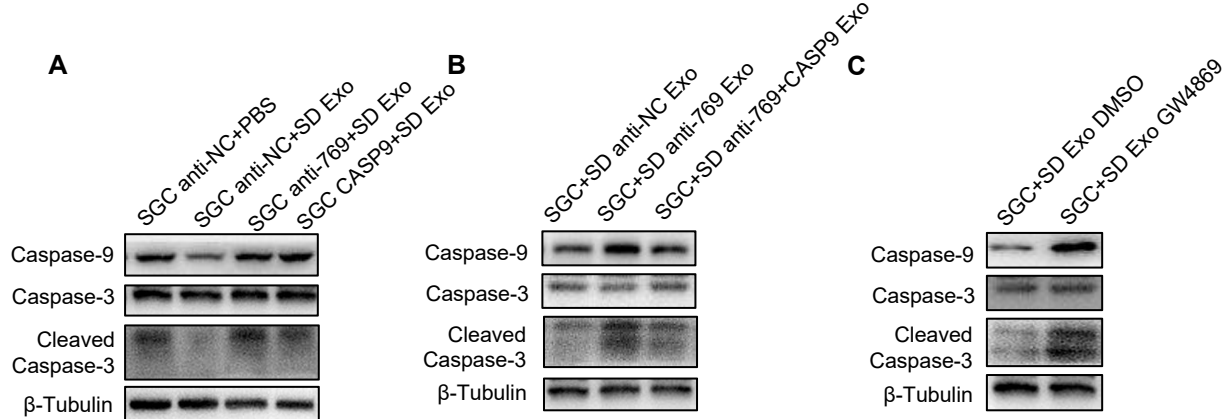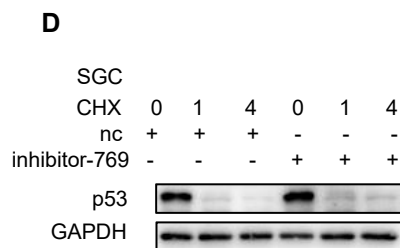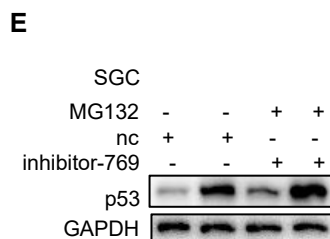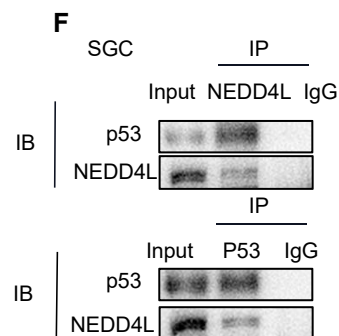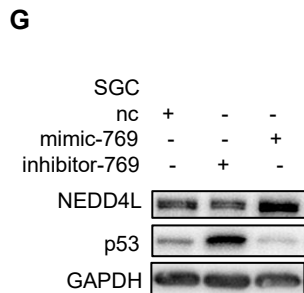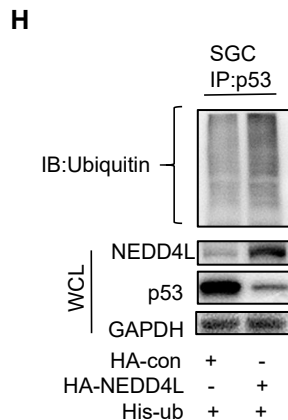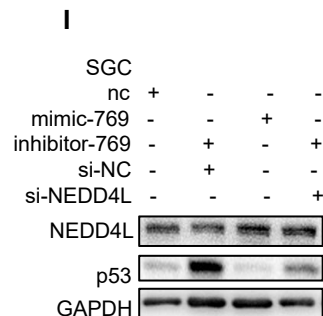

**A**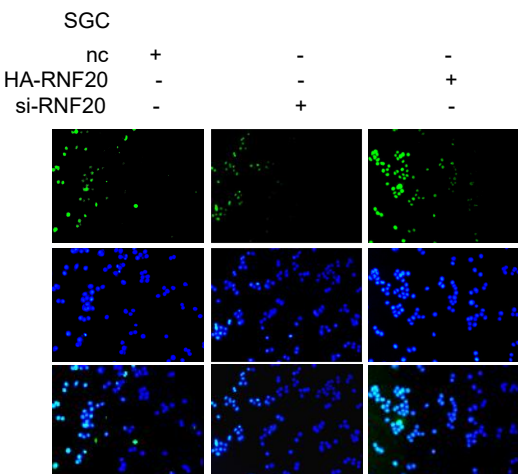**B**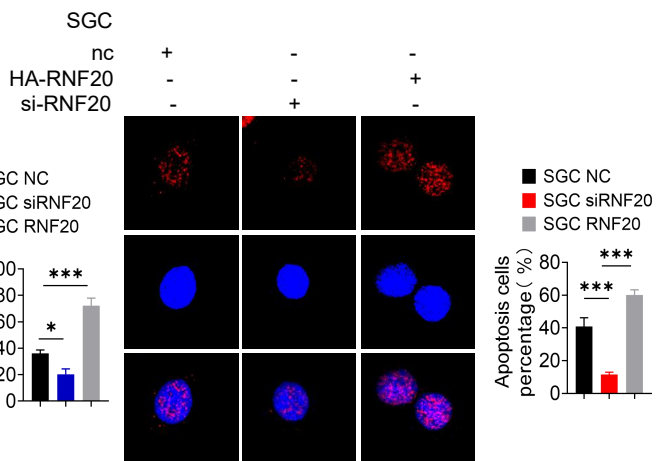**C**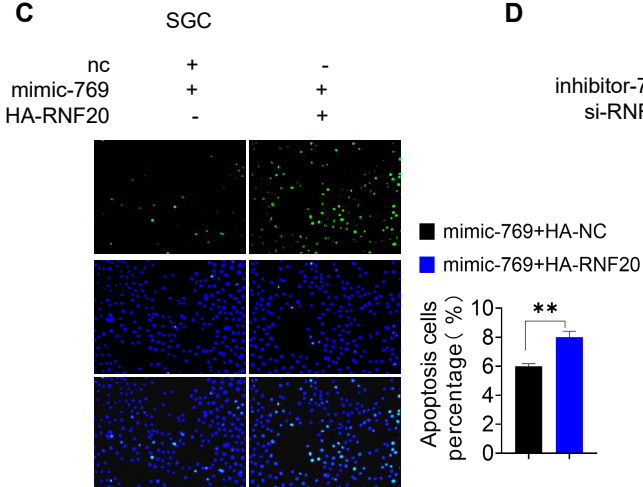**D**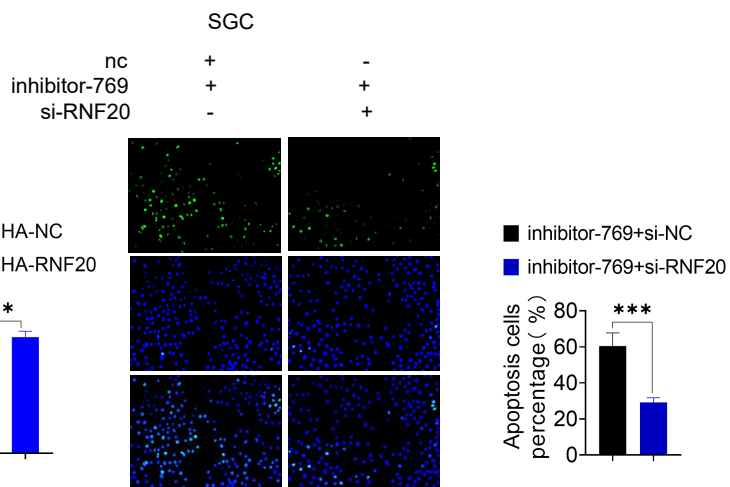

Supplement: Supplementary file 4 — Figure S4‐S5 [file CTM2-12-e780-s006.pdf]

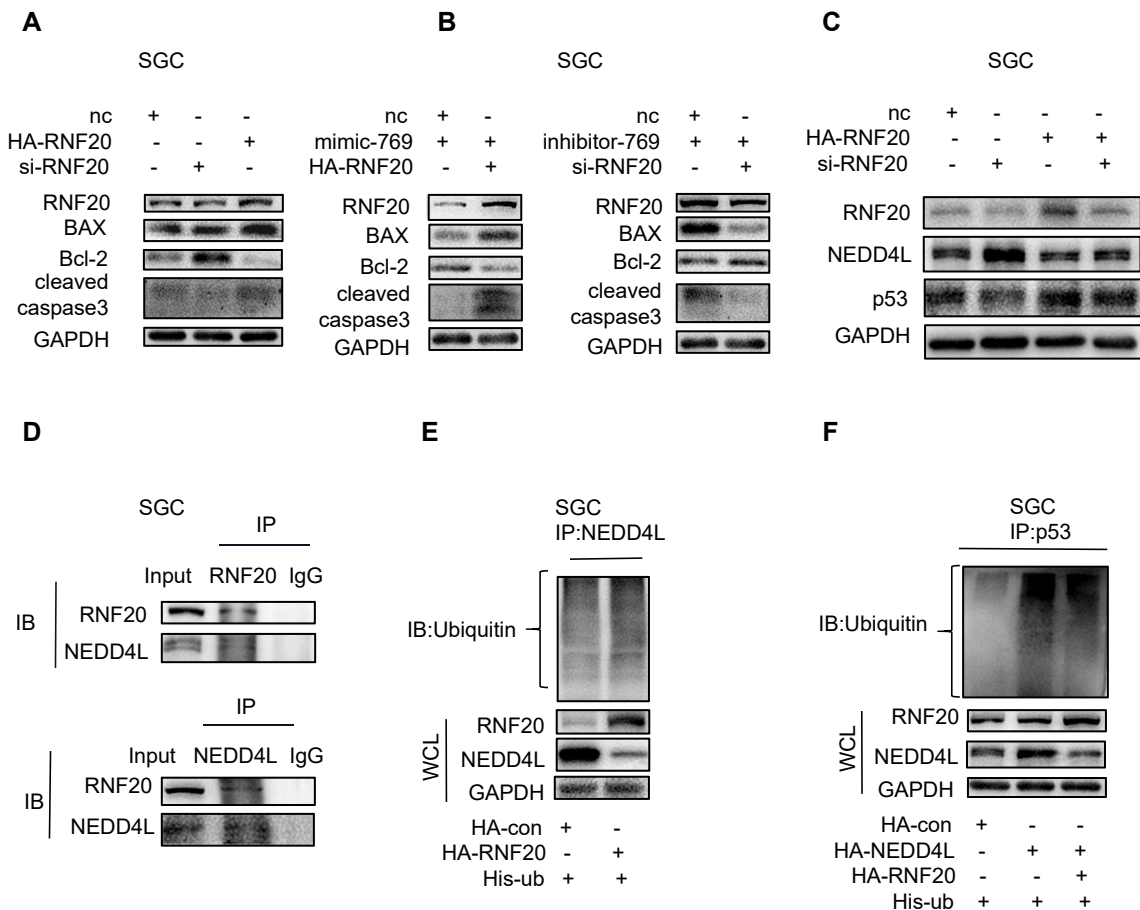

Supplement: Supplementary file 5 — Figure S6 [file CTM2-12-e780-s007.pdf]
