# Supplementary material for: Exosome‐transmitted miR‐769‐5p confers cisplatin resistance and progression in gastric cancer by targeting CASP9 and promoting the ubiquitination degradation of p53
Source: Clin Transl Med. 2022 May 6;12(5):e780. doi: 10.1002/ctm2.780 (PMC9076018; doi:10.1002/ctm2.780)

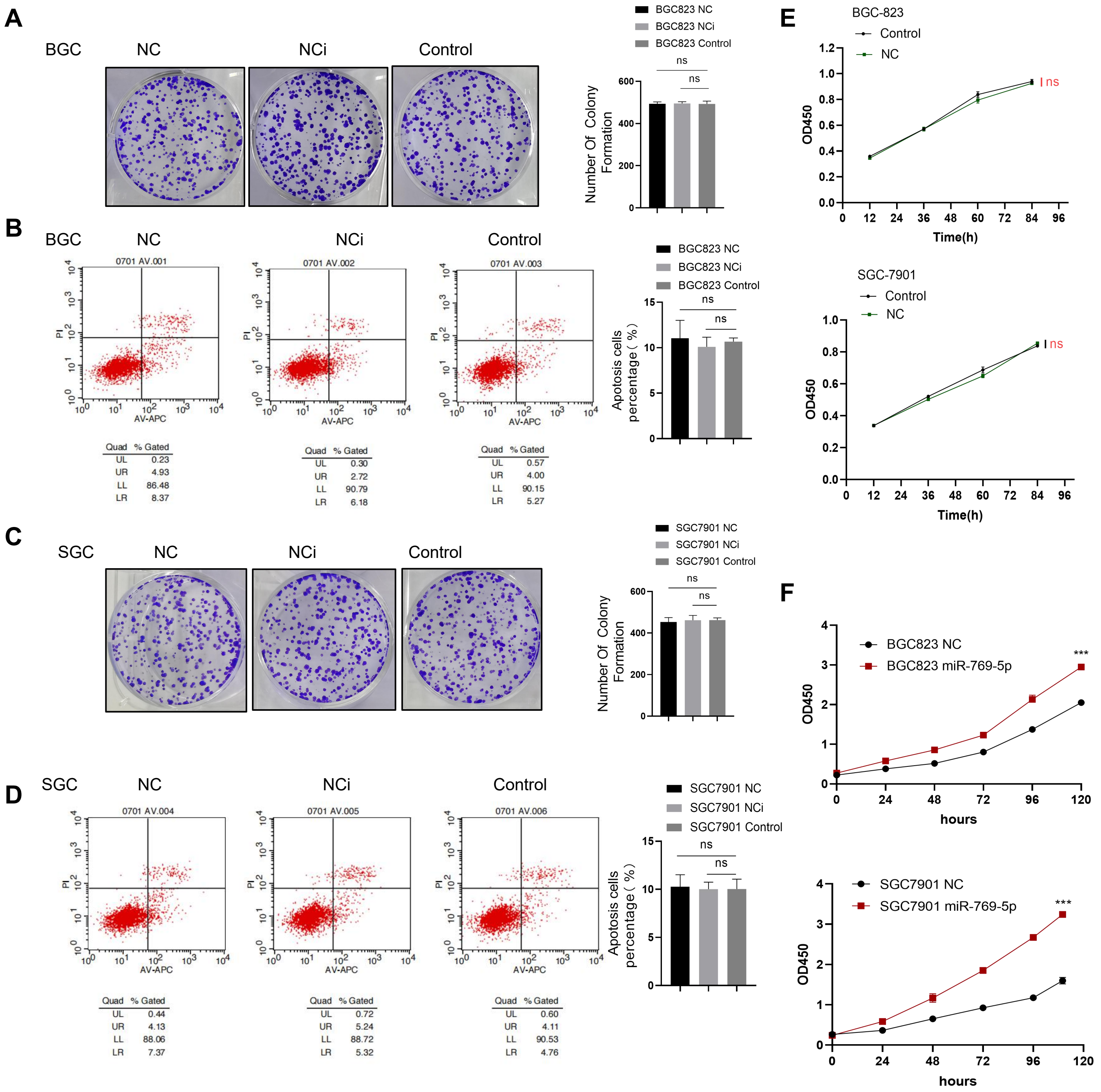

A
BGC
NC
NCi
Control
BGC823 NC
BGC823 NCi
BGC823 Control
ns
ns
Number Of Colony Formation
B
BGC
NC
NCi
Control
0701 AV.001
0701 AV.002
0701 AV.003
PI
AV-APC
Quad % Gated
UL 0.23
UR 4.93
LL 86.48
LR 8.37
Quad % Gated
UL 0.30
UR 2.72
LL 90.79
LR 6.18
Quad % Gated
UL 0.57
UR 4.00
LL 90.15
LR 5.27
BGC823 NC
BGC823 NCi
BGC823 Control
Apoptosis cells percentage (%)
C
SGC
NC
NCi
Control
SGC7901 NC
SGC7901 NCi
SGC7901 Control
Number Of Colony Formation
D
SGC
NC
NCi
Control
0701 AV.004
0701 AV.005
0701 AV.006
PI
AV-APC
Quad % Gated
UL 0.44
UR 4.13
LL 88.06
LR 7.37
Quad % Gated
UL 0.72
UR 5.24
LL 88.72
LR 5.32
Quad % Gated
UL 0.60
UR 4.11
LL 90.53
LR 4.76
SGC7901 NC
SGC7901 NCi
SGC7901 Control
Apoptosis cells percentage (%)
E
BGC-823
Control
NC
OD450
Time(h)
SGC-7901
Control
NC
OD450
Time(h)
F
BGC823 NC
BGC823 miR-769-5p
***
OD450
hours
SGC7901 NC
SGC7901 miR-769-5p
***
OD450
hours

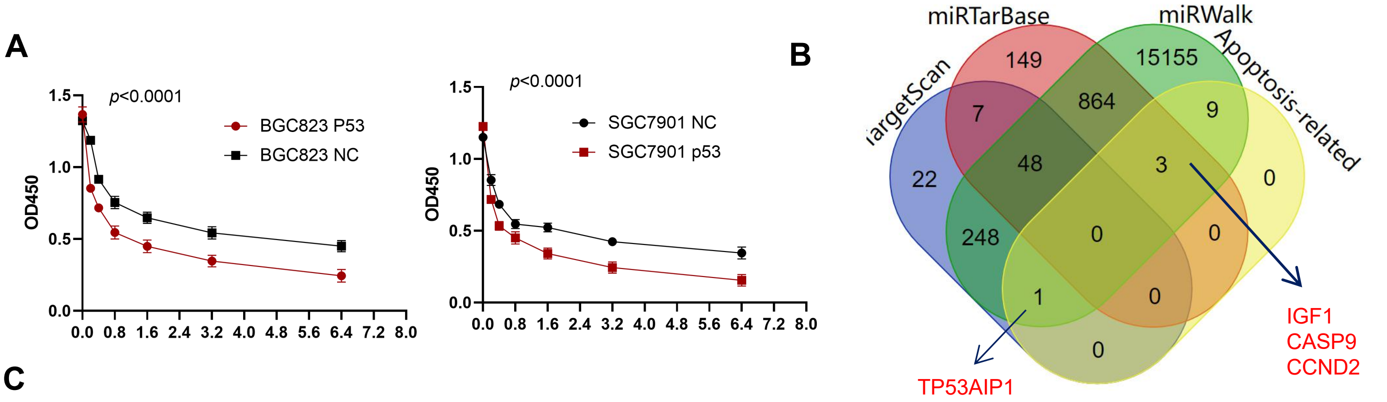

C

KEGG ID	Transcri	Gene ID	Symbol	Gene Annoation	miRNA	KEGG Name	Pvalue of
ko04115	ENST0000	ENSG0000	TSC2	TSC complex subunit 2 [Source:HGNC Sym	hsa-miR-769-5p	p53 signaling pathwa	4.74E-05
ko04115	ENST0000	ENSG0000	TSC2	TSC complex subunit 2 [Source:HGNC Sym	hsa-miR-769-5p	p53 signaling pathwa	4.74E-05
ko04115	ENST0000	ENSG0000	TSC2	TSC complex subunit 2 [Source:HGNC Sym	hsa-miR-769-5p	p53 signaling pathwa	4.74E-05
ko04115	ENST0000	ENSG0000	SERPINE1	serpin family E member 1 [Source:HGNC :	hsa-miR-769-5p	p53 signaling pathwa	4.74E-05
ko04115	ENST0000	ENSG0000	TP53AIP1	tumor protein p53 regulated apoptosis :	hsa-miR-769-5p	p53 signaling pathwa	4.74E-05
ko04115	ENST0000	ENSG0000	TNFRSF10B	TNF receptor superfamily member 10b [S	hsa-miR-769-5p	p53 signaling pathwa	4.74E-05
ko04115	ENST0000	ENSG0000	CDK2	cyclin dependent kinase 2 [Source:HGNC	hsa-miR-769-5p	p53 signaling pathwa	4.74E-05
ko04115	ENST0000	ENSG0000	CDK2	cyclin dependent kinase 2 [Source:HGNC	hsa-miR-769-5p	p53 signaling pathwa	4.74E-05
ko04115	ENST0000	ENSG0000	IGFBP3	insulin like growth factor binding pro	hsa-miR-769-5p	p53 signaling pathwa	4.74E-05
ko04115	ENST0000	ENSG0000	IGFBP3	insulin like growth factor binding pro	hsa-miR-769-5p	p53 signaling pathwa	4.74E-05
ko04115	ENST0000	ENSG0000	IGFBP3	insulin like growth factor binding pro	hsa-miR-769-5p	p53 signaling pathwa	4.74E-05
ko04115	ENST0000	ENSG0000	CDK1	cyclin dependent kinase 1 [Source:HGNC	hsa-miR-769-5p	p53 signaling pathwa	4.74E-05
ko04115	ENST0000	ENSG0000	CDK1	cyclin dependent kinase 1 [Source:HGNC	hsa-miR-769-5p	p53 signaling pathwa	4.74E-05
ko04115	ENST0000	ENSG0000	CDK1	cyclin dependent kinase 1 [Source:HGNC	hsa-miR-769-5p	p53 signaling pathwa	4.74E-05
ko04115	ENST0000	ENSG0000	CDK1	cyclin dependent kinase 1 [Source:HGNC	hsa-miR-769-5p	p53 signaling pathwa	4.74E-05
ko04115	ENST0000	ENSG0000	CDK1	cyclin dependent kinase 1 [Source:HGNC	hsa-miR-769-5p	p53 signaling pathwa	4.74E-05

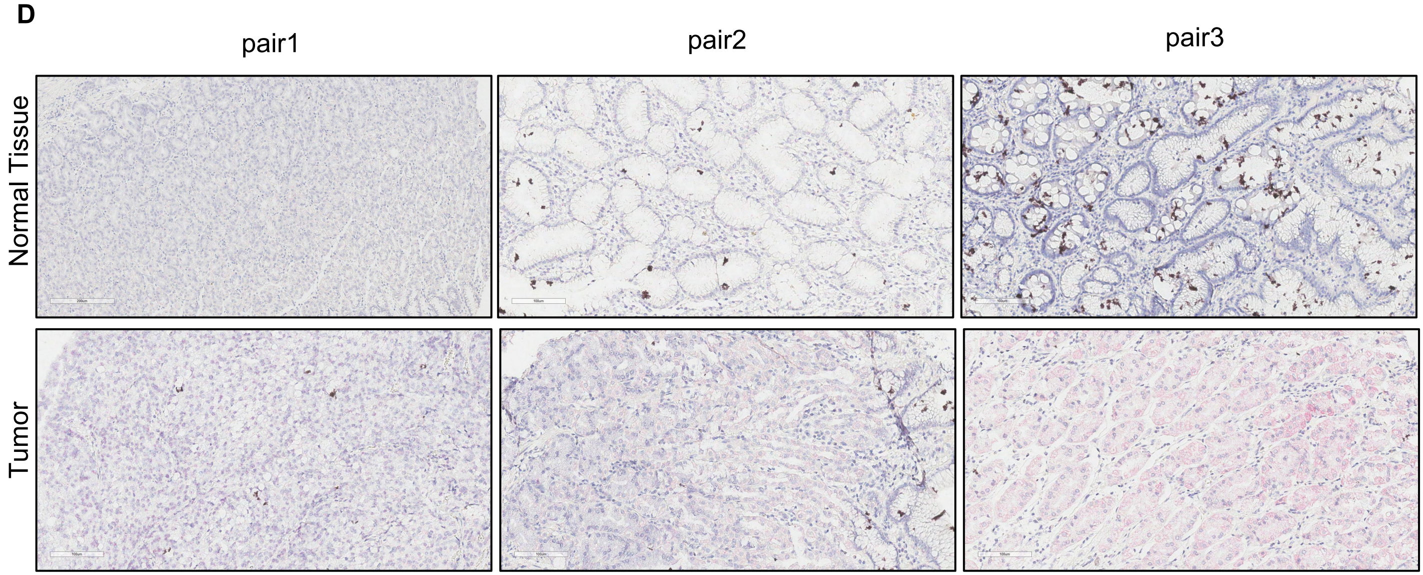

Supplement: Supplementary file 6 — Figure S7‐S8 [file CTM2-12-e780-s008.pdf]
