# Supplementary material for: Exosome‐transmitted miR‐769‐5p confers cisplatin resistance and progression in gastric cancer by targeting CASP9 and promoting the ubiquitination degradation of p53
Source: Clin Transl Med. 2022 May 6;12(5):e780. doi: 10.1002/ctm2.780 (PMC9076018; doi:10.1002/ctm2.780)
Supplement: Supplementary file 7 — Supporting Information [file CTM2-12-e780-s004.pdf]

Supplementary Materials and Methods

Patient tissue and blood samples

Samples for cancer patients, including tissue and plasma specimens, were collected from the First Affiliated Hospital of Nanjing Medical University. Blood samples (serum) from 19 cisplatin-resistant patients and 41 cisplatin-sensitive patients were collected and stored at −80 ° C. Other samples of 150 cases (75 pairs of GC tumor and normal tissuess) were embedded with 75 paraffin and analyzed by tissue microarray. Clinicopathological features, including age, sex, tumor site, tumor size, differentiation grade, Lauren classification, TNM stage (American Joint Committee on Cancer classification, AJCC), and lymphatic invasion, were also collected and analyzed **(Table 1).** This study was approved by the Ethics Committee of the First Affiliated Hospital of Nanjing Medical University. All patients signed an informed consent.

Cell culture and treatment

The HEK-293T cell line was purchased from Type Culture Collection of the Chinese Academy of Sciences (Shanghai, China). Gastric cancer BGC823, SGC7901 cell lines, cisplatin-resistant BGC823/ DDP, and SGC7901/DDP cells were a kind gift from Professor Jianwei Zhou (School of Public Health, Nanjing Medical University). All cell lines were cultured in RPMI 1640 media (Gibco, Carlsbad, CA, USA) containing 10% fetal bovine serum (FBS) (ScienCell, CA, USA) and supplemented with 100

μg/ml streptomycin, 100 U/ml penicillin in a humidified atmosphere containing 5% CO_2 at 37 °C. BGC823/ DDP and SGC7901/ DDP cells were cultured in a medium maintained with 0.5 μg/ml cisplatin (First Affiliated Hospital of Nanjing Medical University). Before the experiments, cell were cultured in a drug-free medium for at least 7 days. Cycloheximide (CHX)(Sigma-Aldrich, MO, USA) and MG132 (Selleck Chemicals, USA) were used at the indicated concentrations.

Exosome isolation and characterization

Cell culture supernatant was collected after being washed with PBS and incubated with freshly prepared complete medium containing exosome-free FBS for 48h. Exosomes were isolated from the conditioned medium by differential centrifugation. Conditioned medium was centrifuged at 300 g for 10 min and then at 2,000 g for 20 min at 4 ° C. The supernatant was then passed through a 0.22- μ m filter (Millipore, Burlington, MA, USA) to remove shedding vesicles and other vesicles larger in size. Finally, the supernatant was centrifuged at 110,000 × g for 70 min. Pelleted exosomes were resuspended in PBS and collected by ultracentrifugation again at 100,000 g for 90 min (all steps were performed at 4 ° C). Exosomes were collected from the pellet and resuspended in 100 μ L of PBS and subjected to several experiments. The fractionation and purification of exosomes from conditioned media (CMs) and blood serum were collected by ultracentrifugation (Beckman Coulter) and ExoQuick Exosome Precipitation Solution (SBI, CA, USA) respectively. Exosomes were then identified by Transmission Electron Microscope (TEM) (Philips TECNAI

20, Netherland), and their particle morphology and size were analyzed. The concentration and number of exosomes were detected by nanoparticle tracking analysis (NTA). Exosome protein markers were identified by Western blot assay and flow cytometry analysis (FACS Calibur, BD Biosciences, USA) .

Human serum exosomes were obtained with ExoQuick Exosome Precipitation Solution (SBI, CA, USA) following the user manual. Briefly, serum was collected and centrifuged at 3000 × g for 15 minutes. Then add the 63 ul ExoQuick Exosome Precipitation Solution to 250 ul supernatant and refrigerate the mixture 30 minutes serum at 4°C. After centrifugation at 1500 × g for 30 minutes, resuspend exosome pellet in 100 μl using sterile 1× PBS.

PKH26 Staining for Exosomes

The isolated exosomes were labeled with PKH26 Red Fluorescent Cell Linker Kits (Sigma). Exosomes were first resuspended in 100 μL Diluent C. A dye solution (4 × 10−6 M) was prepared by adding 0.4 μL PKH26 ethanolic dye solution to 100 μL Diluent C. The 100 μL exosome suspension was then mixed with the 100 μL dye solution by pipetting. After incubating the cell and dye suspension for 5 min with periodic mixing, the staining was stopped by adding 200 μL serum and incubating for 1 min. The stained exosomes were finally washed twice with 1× PBS, and they were resuspended in a fresh sterile conical polypropylene tube.

Lentiviral, plasmid, and microRNA mimics/inhibitors package and cell transfection

The lentivirus encoding miR-769-5p overexpression or knockdown and negative control (769, NC, anti-769, anti-NC) were designed and produced by GENECHEM (Shanghai, China). The lentivirus were added to BGC823 BGC823/DDP, SGC7901 and SGC7901/DDP cells respectively and stable cell lines were obtained by selection with puromycin (Sigma-Aldrich, MO, USA). The infection efficiency was confirmed by fluorescence microscopy and real-time quantitative RT-PCR (qRT-PCR). pcDNA3.1 vector containing CASP9-wt, CASP9-mut, RNF20-wt or RNF20-mut, and a control vector were purchased from GENECHEM (Shanghai, China). miR-769-5p mimics, inhibitor and control, Cy3-miR-769-5p mimics and control were produced by GenePharma (Shanghai, China). Plasmids and miRNA mimics or inhibitors were transfected into cells with Lipofectamine 3000 (Invitrogen) according to the manufacturer's instructions. The siRNAs and controls were designed and synthesized by RiboBio (Guangzhou, China). The siRNAs were transfected into the cells by DharmaFECT4 (Dharmacon, IL, USA); all sequences are listed in **Additional file 2 Table S1**.

RNA extraction and quantitative RT-PCR

Total cellular and exosomal RNA was extracted from exosomes, co-cultured cells or GC cells, and frozen xenograft tumor tissues using TRIzol reagent (Invitrogen, CA, USA). Isolated RNA was used for the reverse transcription reaction with HiScript Q RT SuperMix for qPCR (Vazyme, Jiangsu, China). Quantitative RT-PCR was carried out with SYBR Green PCR Master Mix (Vazyme) using an ABI Prism 7900

Sequence detection system (Applied Biosystems, Canada). The relative expression of miR-769 was normalized to U6 levels, and CASP9, RNF20, p53 mRNA expression were normalized to GAPDH by qPCR using Power SYBR Green (Takara, Dalian, China). Data were calculated by the2 (−ΔΔCT) method. The related primers are synthesized by Ribobio (Guangzhou, China) and listed in **Additional file 2: Table S2**.

Dual-luciferase reporter assays

293T cells (3 × 104 cells per well) were seeded onto 24-well plates 1 day before transfection and were co-transfected by Lipofectamine™ 3000 (Invitrogen, USA) with luciferase reporter (200 ng per well) using pmiR-REPORT™ luciferase vectors (pmirGLO) containing wild-type or mutant 3`-UTR of CASP9 and RNF20 and miR-769-5p mimics or miR-769-5p mimic-NC to examine the miRNA binding ability. The cells were washed and lysed with the passive lysis buffer from the Dual-Luciferase Reporter Assay System (Promega Corp). About 24 h later, a Dual-Luciferase Reporter Assay kit (Promega, USA) was used to measure the luciferase and renilla activity of these samples according to the manufacturer's instructions. Relative luciferase activity was first normalized with Renilla luciferase activity and then compared with those of the respective control. Wild-type and mutated CASP9 or RNF20 3′ UTRs were synthesized and inserted into the p-MIR-REPORT plasmid by Genechem, Shanghai, China.

Colony formation assay

GC cells (500 cells/well in six-well) were performed to detect the proliferation capacity. After incubation at 37 °C, 5% CO2 for two weeks, the plates were washed with PBS, fixed with 4% paraformaldehyde, stained with 0.1% crystal violet, washed three times with water, and analyzed. The assay was repeated three times in duplicate, and the numbers of colony formation counted.

Cell viability assay

Cells (1 × 104/well) were seeded in 96-well plates and treated with cisplatin from 0.2 to 6.4 μg/ml for 24 h. A CCK-8 assay was performed to detect cells viability using a Cell Counting Kit 8 (Dojindo, Japan) and a OD450 nm (Synergy4; BioTek, Winooski, VT, USA). Based on protocols of CCK-8 kits cells were seeded, cultured for 24 h, and further cultured in 100 μL medium with 10 μL CCK-8 reagent. Absorbance at 450 nm was determined using a Multiscan FC plate reader (Thermo Fisher).

Cell Migration Assay

The migratory capacity of GCs was tested by using a Transwell Boyden Chamber (6.5 mm, Costar) with polycarbonate membranes (8-μm pore size) on the bottom of the upper compartment. A total of 2 × 104 cells was suspended in serum-free media. Meanwhile, the lower chambers were loaded with 0.5 mL RPMI1640 containing 5% FBS, and the plates containing Transwell inserts were incubated. After incubation at 37 °C, 5% CO2 for 12 h, the upper chamber was cleaned with a cotton swab, and the lower chamber was washed with PBS. The cells that penetrated through the membrane were fixed with 90% ethanol for 15 min at room temperature, stained with

0.1% crystal violet solution, washed three times with water, and imaged by Inversion Microscope (Zeiss, Germany). The assay was repeated three times in duplicate. We obtained images of migrated cells by using a photomicroscope, and we quantified cell migration by blind counting with five fields per chamber.

Apoptosis assay

The flow cytometry analysis was performed by Annexin V-APC/PI Apoptosis Detection Kit (Vazyme, Jiangsu, China) according to the manufacturer's instructions. The cells were analyzed with a BD FACS Calibur flow cytometer using CellQuest Pro software (FACS Calibur, BD Biosciences, USA).

TUNEL assay

GC cells were fixed with paraformaldehyde for 30 min on ice. Then, terminal deoxynucleotidyl transferase dUTP nick end labeling (TUNEL) kit was used according to the manufacturer's instructions (TUNEL BrightGreen Apoptosis Detection Kit, Vazyme, Jiangsu, China) and DAPI (4′,6-diamidino-2-phenylindole) was used for nuclear staining. TUNEL-positive areas were quantified under an Olympus FSX100 microscope (Olympus, Tokyo, Japan).

Fluorescence assay

4′,6-diamidino-2-phenylindole (DAPI) (Invitrogen, USA) was used for cell nuclear staining. Rhodamine-conjugated secondary antibody (Cell Signaling Technology, USA) for γ-H2AX (1:250, Abcam, ab81299) protein and DAPI for nuclear staining.

The slides were visualized for immunofluorescence with a laser scanning microscope (Zeiss, Germany).

Western blot, immunohistochemistry (IHC), and immunoprecipitation (IP) assay

Cell or tissue samples were lysed by RIPA buffer mixed with protease and phosphatase inhibitor cocktails. Serum proteins were extracted with Serum Protein Extraction Kit (Qcheng Bio, China). The proteins were then separated by 10% SDS-PAGE and transferred onto PVDF membranes. Western blot assays were performed according to previously reported data [1]

The immune-complexes were detected with ECL Western Blotting Substrate (Thermo Fisher) and visualized with BIO-RAD (BIO-RAD Gel Doc XR+, USA). Immunohistochemistry and immunoprecipitation were done as previously reported [2]. Positive cells were counted in five random fields per slide. Primary antibodies and appropriate secondary antibodies used for the experiments are listed: TSG101 (1:1000, Abcam, ab125011), Calnexin (1:1000, Abcam, ab92573), CD81 (1:1000, Proteintech, 66866-1-Ig), CD63 (1:1000, Abcam, ab134045), γ -H2AX (1:250, Abcam, ab81299), caspase-9 (1:1000, CST, # 9504S), caspase-3 (1:1000, CST, # 9662), cleaved caspase-3 (1:1000, CST, # 9661), BAX (1:10000, Proteintech, 50599-2-Ig), Bcl-2 (1:1000, CST, #3498), p53 (1:5000, Proteintech, 10442-1-AP), NEDD4L (1:5000, Proteintech, 13690-1-AP), RNF20(1:1000, Proteintech, 21625-1-AP), Ubiquitin(1:1000, CST, # 3936S), β-actin (1:1000, Beyotime, AF0003), GAPDH (1:1000, Beyotime, AF0006). Incubation with the goat anti-rabbit secondary

antibody (1:1000, Beyotime, A0208) or the goat anti-mouse secondary antibody (1:1000, Beyotime, A0216).

RNA in situ hybridization (ISH)

BaseScope™ Reagent Kit v2-RED (Advanced Cell Diagnostics, CA, USA) was used for ISH following the user manual. RNA in situ hybridization (ISH) was performed according to previously reported data . Standard RNAScope protocols were used according to manufacturer's instructions and were performed according to previously reported data [3]. The following probes were used: miRNAscope Probe - SR-hsa-miR-769-5p-S1 (ACD; 1029501-S1), miRNAscope Positive Control Probe - SR-RNU6-S1 (ACD; 727871-S1), miRNAscope Negative Control Probe - SR-Scramble-S (ACD; 727881-S1).

A nude mouse model

a. 4-week-old (BALB/c) were obtained from Model Animal Research Center Of Nanjing University, China. All the animals were housed in an environment with a temperature of 22 ± 1 °C, relative humidity of 50 ± 1%, and a light/dark cycle of 12/12 hr and had access to water and food at libitum. All animal studies (including the mice euthanasia procedure) were done in compliance with the regulations and guidelines of Nanjing Medical University institutional animal care and conducted according to the AAALAC and the IACUC guidelines (IACUC-1902006). Forty 4-week-old (BALB/c) male nude mice were randomly divided into two groups (20 mice in each group): BGC823+PBS and BGC823+BD EXO group. Briefly, 5×10^6

BGC823 cells (100μL) were subcutaneously injected into the right flank of nude mice. When the average volume of nude mice reached approx. 50mm^3, one group was intratumorally injected with BGC/DDP EXO (200ug/100μL cells per mouse) once every two days. When the tumor volume was 150-200mm^3, each group were divided into two groups (10 mice in each group): BGC823+PBS+PBS, BGC823+PBS+DDP, BGC823+BD EXO+PBS and BGC823+BD EXO+DDP group, one group (BGC823+PBS+DDP, BGC823+BD EXO+DDP) was intraperitoneally injected with DDP (4mg/kg per mouse) every three days, and the other group (BGC823+PBS+PBS, BGC823+BD EXO+PBS) was injected with normal saline as the control group.

b. Forty 4-week-old (BALB/c) male nude mice were randomly divided into two groups (20 mice in each group): BGC NC and BGC 769. BGC823 cells with stable overexpression of miR-769-5p (BGC 769) and control cells (BGC NC) (5 × 10^6/100μL cells per mouse) were subcutaneously injected into the right flank of nude mice. When the average volume of nude mice was about 150-200mm^3, each group was divided into two groups: BGC NC+PBS, BGC 769+PBS, BGC NC+DDP and BGC 769+DDP. One group (BGC/DDP anti769+DDP, BGC/DDP anti-NC+DDP) was intraperitoneally injected with DDP according to the standard of 4mg/kg every three days, and the other group (BGC NC+PBS, BGC 769+PBS) was intraperitoneally injected with normal saline as control.

c. Forty 4-week-old (BALB/c) male nude mice were randomly divided into two groups: BGC/DDPanti-769 and BGC/DDP anti-NC, with 20 mice in each group.

BGC/DDP cells and control cells with stable knockdown expression of miR-769-5p (5×10^6/100 μ L cells per mouse) were injected subcutaneously into the right flank of nude mice. When the average volume of nude mice was about 150-200mm^3, each group was divided into two groups on average: BGC/DDPanti-769+PBS, BGC/DDP anti-NC+PBS, BGC/DDP anti769+DDP and BGC/DDP anti-NC+DDP. One group (BGC/DDP anti769+DDP, BGC/DDP anti-NC+DDP) was intraperitoneally injected with DDP according to the standard of 4mg/kg every three days, and the other group (BGC/DDPanti-769+PBS, BGC/DDP anti-NC+PBS) was intraperitoneally injected with normal saline as control.

Three weeks later, mice were sacrificed, and tumor tissues were prepared for histological examination: H&E staining, Western blot, and IHC assays. Tumor volume was measured using the following formula: $Tumor\ volume\ (mm^3) = 0.5 \times width^2 \times length.$

Statistical analysis

Statistical data were expressed as mean ± SD. One-way analysis of variance was used for three groups and more than three groups. All of the statistical analyses were assessed by software SPSS version 13.0 (SPSS, Chicago, IL, USA)and GraphPad Prism (GraphPad Software, Inc., SanDiego, CA, USA) software, comparisons among groups were done by the independent sample two-sided Student t-test. The ANOVA was performed to evaluate the statistical differences among groups. P- value of 0.05 or less was considered as statistical significance.

References:

1. Zhou, J., Ye, J., Zhao, X., Li, A., and Zhou, J. (2008). JWA is required for arsenic trioxide induced apoptosis in HeLa and MCF-7 cells via reactive oxygen species and mitochondria linked signal pathway. *Toxicol Appl Pharmacol* 230: 33-40.
2. Qiu, D., et al. (2018). RNF185 modulates JWA ubiquitination and promotes gastric cancer metastasis. *Biochim Biophys Acta Mol Basis Dis* 1864: 1552-1561.
3. Xie, M., et al. (2020). Exosomal circSHKBP1 promotes gastric cancer progression via regulating the miR-582-3p/HUR/VEGF axis and suppressing HSP90 degradation. *MOL CANCER* 19: 112.
